# Ebola Virus Binding to Tim-1 on T Lymphocytes Induces a Cytokine Storm

**DOI:** 10.1128/mBio.00845-17

**Published:** 2017-09-26

**Authors:** Patrick Younan, Mathieu Iampietro, Andrew Nishida, Palaniappan Ramanathan, Rodrigo I. Santos, Mukta Dutta, Ndongala Michel Lubaki, Richard A. Koup, Michael G. Katze, Alexander Bukreyev

**Affiliations:** a Department of Pathology, The University of Texas Medical Branch, Galveston, Texas, USA; b Department of Microbiology and Immunology, The University of Texas Medical Branch, Galveston, Texas, USA; c Galveston National Laboratory, The University of Texas Medical Branch, Galveston, Texas, USA; d The University of Texas Medical Branch, Galveston, Texas, USA; e Department of Microbiology, University of Washington, Seattle, Washington, USA; f Washington National Primate Research Center, Seattle, Washington, USA; g Immunology Laboratory, Vaccine Research Center, National Institute of Allergy and Infectious Diseases, National Institutes of Health, Bethesda, Maryland, USA; Indiana University Bloomington

**Keywords:** cytokine storm, T lymphocytes, transcriptome, cytokines, Ebola virus, viral pathogenesis

## Abstract

Ebola virus (EBOV) disease (EVD) results from an exacerbated immunological response that is highlighted by a burst in the production of inflammatory mediators known as a “cytokine storm.” Previous reports have suggested that nonspecific activation of T lymphocytes may play a central role in this phenomenon. T-cell immunoglobulin and mucin domain-containing protein 1 (Tim-1) has recently been shown to interact with virion-associated phosphatidylserine to promote infection. Here, we demonstrate the central role of Tim-1 in EBOV pathogenesis, as Tim-1^−/−^ mice exhibited increased survival rates and reduced disease severity; surprisingly, only a limited decrease in viremia was detected. Tim-1^−/−^ mice exhibited a modified inflammatory response as evidenced by changes in serum cytokines and activation of T helper subsets. A series of *in vitro* assays based on the Tim-1 expression profile on T cells demonstrated that despite the apparent absence of detectable viral replication in T lymphocytes, EBOV directly binds to isolated T lymphocytes in a phosphatidylserine–Tim-1-dependent manner. Exposure to EBOV resulted in the rapid development of a CD4^Hi^ CD3^Low^ population, non-antigen-specific activation, and cytokine production. Transcriptome and Western blot analysis of EBOV-stimulated CD4^+^ T cells confirmed the induction of the Tim-1 signaling pathway. Furthermore, comparative analysis of transcriptome data and cytokine/chemokine analysis of supernatants highlight the similarities associated with EBOV-stimulated T cells and the onset of a cytokine storm. Flow cytometry revealed virtually exclusive binding and activation of central memory CD4^+^ T cells. These findings provide evidence for the role of Tim-1 in the induction of a cytokine storm phenomenon and the pathogenesis of EVD.

## IMPORTANCE

Ebola virus infection is characterized by a massive release of inflammatory mediators, which has come to be known as a cytokine storm. The severity of the cytokine storm is consistently linked with fatal disease outcome. Previous findings have demonstrated that specific T-cell subsets are key contributors to the onset of a cytokine storm. In this study, we investigated the role of Tim-1, a T-cell-receptor-independent trigger of T-cell activation. We first demonstrated that Tim-1-knockout (KO) mice survive lethal Ebola virus challenge. We then used a series of *in vitro* assays to demonstrate that Ebola virus directly binds primary T cells in a Tim-1–phosphatidylserine-dependent manner. We noted that binding induces a cytokine storm-like phenomenon and that blocking Tim-1–phosphatidylserine interactions reduces viral binding, T-cell activation, and cytokine production. These findings highlight a previously unknown role of Tim-1 in the development of a cytokine storm and “immune paralysis.”

## INTRODUCTION

The recent Ebola virus (EBOV) outbreak in West Africa has resulted in more than 27,000 infections with more than 11,000 fatalities (1). While the efficacies of several EBOV candidate vaccines and therapeutic strategies are currently being assessed, supportive care remains the primary method of treatment (2). Moreover, despite a moderate efficiency, EBOV candidate vaccines are associated with harmful side effects, including high levels of inflammation and lymphopenia (3–6). Unraveling the complex and multiple mechanisms employed by EBOV that lead to rapid disease progression remains critical to the development of postexposure therapeutic interventions.

Copious EBOV replication within dendritic cells (DCs) and the monocyte-macrophage lineage (7, 8) renders both the innate and adaptive immune responses ineffective shortly following infection. Several groups, including ours, have shown that EBOV-infected DCs are incapable of functional maturation (9–11), resulting in an impaired ability to activate antigen-specific T-lymphocyte responses (12, 13). A characteristic feature of EBOV infections is lymphopenia, which is observed in both humans and experimentally infected nonhuman primates (NHP) (8, 14–20). Lymphopenia is typically observed in EBOV patients who succumb to disease, whereas survivors have been shown to maintain CD3^+^ T-lymphocyte populations throughout the course of disease (21, 22). Strikingly, lymphopenia occurs despite the inability of EBOV to infect lymphocytes (14, 23).

Several clinical and experimental studies have correlated the massive burst in immunological mediators, known as a cytokine storm, with morbidity and mortality associated with influenza, bacterial sepsis, and viral hemorrhagic fever diseases (24–29). Cytokine storm has also been implicated as a central factor contributing to EBOV disease (EVD) (21). A cytokine storm in response to viral infections is characterized by induction of both pro- and anti-inflammatory responses. Inflammatory mediators induced during a severe cytokine storm usually include interferons (IFNs), tumor necrosis factors (TNFs), interleukins (ILs), and chemokines (24, 30, 31). Overall, more than 150 cytokines have been proposed to contribute to the development of a cytokine storm, which, in combination with a relative redundancy of cytokine/chemokine signaling, has been highly detrimental to the development of effective treatments (24).

The precise mechanisms of induction of cytokine storm are largely unknown. However, it is known that numerous unrelated viruses and bacteria trigger cytokine storm by engagement of T-cell receptor (TCR) and CD28 or by activation of pathogen-associated molecular pattern (PAMP) recognition pathways, such as Toll-like receptors (32–35). Furthermore, it has been suggested that following the release of proinflammatory mediators by virus-infected macrophages, T lymphocytes respond by further contributing to the accumulation of the proinflammatory mediators which contribute to the immunopathology associated with the onset of a cytokine storm and further activate macrophages (36, 37). This suggests that the development of a cytokine storm is the result of a feed-forward loop.

Monocytes and DCs are among primary target cells of EBOV (7); despite infection, their ability to secrete cytokines and chemokines and undergo maturation is limited (9–11), which further limits their capacity to activate an adaptive immune response. This suggests that other cell types may contribute to the mass production of pro- and anti-inflammatory mediators during EBOV infection, although the identity of these cells and the mechanism by which they contribute to the development of a cytokine storm remain to be elucidated.

The membrane protein Niemann-Pick C1 (NPC1) has been identified as an endosomal receptor that is required for EBOV entry into target cells (38, 39). Recently, the T-cell immunoglobulin and mucin domain-containing protein 1 (Tim-1) was identified as an EBOV attachment factor (40). Both human and murine macrophages have been shown to express Tim-1 and phagocytose apoptotic bodies via interaction with its membrane-associated phosphatidylserine (PS) (41). Similarly, EBOV entry *in vitro* was shown to be dependent on virion-associated PS (42). Due to the roles of Tim-1 in enveloped-virus binding (40, 42) and immunomodulation of the inflammatory responses following activation of its signaling pathway (43, 44), we investigated the role of Tim-1 in the pathogenesis of EVD *in vivo* and extended our analysis *ex vivo* to determine what effects, if any, EBOV may have directly on CD4^+^ T cells.

## RESULTS

### Tim-1 is involved in the pathogenesis of EVD.

**FIG 1:**
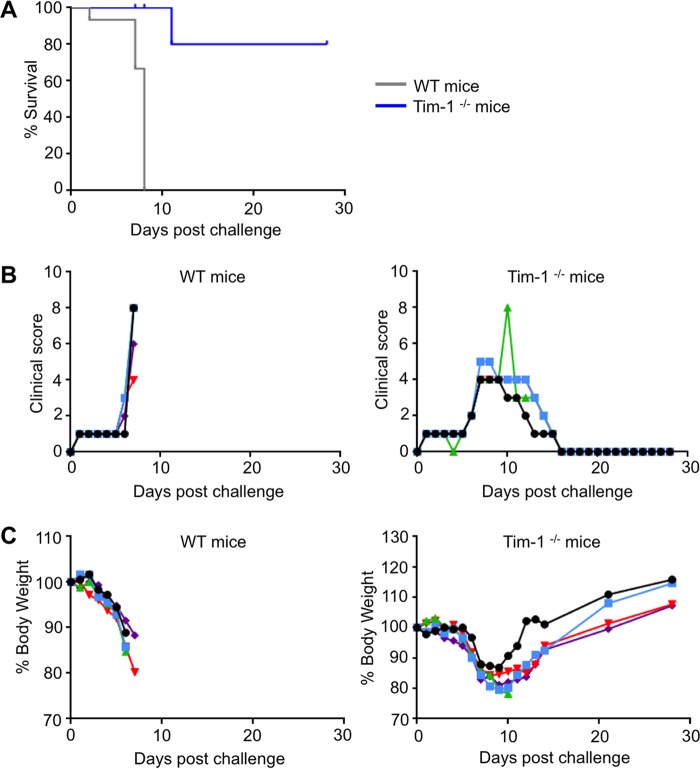
Role of Tim-1 in pathogenesis of EBOV disease. Wild-type (WT) mice and Tim-1^−/−^ mice were infected with mouse-adapted EBOV at 5 animals per group. (A) Survival curves, *P* < 0.0001 (Mantel-Cox test). (B) Clinical scores assigned as described in Materials and Methods. (C) Changes of mouse body weight in percent.

In order to determine the extent to which Tim-1 is associated with EVD pathogenesis, Tim-1^−/−^ mice were infected with a mouse-adapted strain of EBOV (45). One hundred percent of control, wild-type (WT) mice died or were moribund and euthanized as required by protocol by day 8 postinfection (Fig. 1A). Strikingly, 80% of Tim-1-knockout (KO) mice survived, with only one fatality occurring at day 11 postinfection. An increase of illness scores (Fig. 1B) and weight loss (Fig. 1C) was observed in both groups; however, the surviving Tim-1^−/−^ mice completely recovered by day 16 postinfection. Based on these findings and previous reports regarding the known functions of Tim-1, we conducted in-depth analysis to determine whether the absence of Tim-1 altered the inflammatory response.

**FIG 2:**
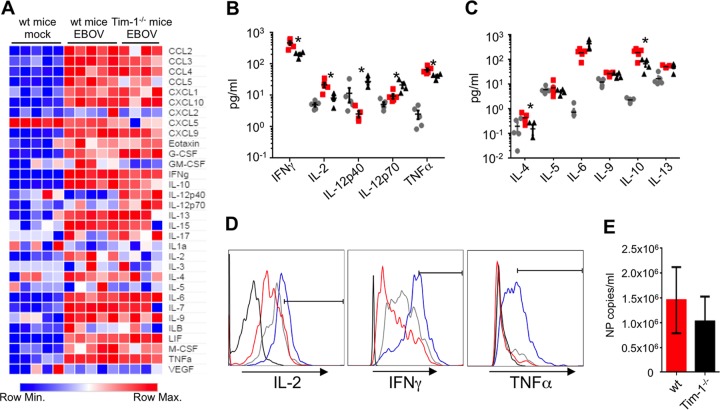
Tim-1^−/−^ mice exhibit reduced Th1/2 responses. Serum cytokines/chemokines, CD4^+^ T-cell functional responses, and plasma viremia levels were analyzed 6 days following EBOV infection. (A) Heat map representing global variations in cytokine/chemokine responses in wild-type C57BL/6J mice and Tim-1^−/−^ as determined by multiplex analysis. G-CSF, granulocyte colony-stimulating factor; LIF, leukemia inhibitory factor; M-CSF, macrophage colony-stimulating factor; VEGF, vascular endothelial growth factor. (B and C) Graphical representation of Th1-associated (B) and Th2-associated (C) cytokines. Gray circles, mock-infected wild-type mice; red squares, EBOV-infected wild-type mice; black triangles, Tim-1^−/−^ EBOV-infected mice. (D) Intracellular cytokine staining for IL-2, IFN-γ, and TNF-α in gated CD4^+^ CD3^+^ T cells. Black line, isotype control, an EBOV-infected wild-type mouse; red line, a mock-infected wild-type mouse; gray line, an EBOV-infected wild-type mouse; blue line, an EBOV-infected Tim-1^−/−^ mouse. (E) Plasma viremia levels were determined by quantitative PCR. Histograms of cells are representative of individual mice within each group. Plasma viremia is shown by the mean number ± SE of NP copies from 5 wild-type C57BL/6J mice and 4 Tim-1^−/−^ mice infected with mouse-adapted EBOV. Asterisks denote statistical significances between the mean averages for wild-type mice and Tim-1^−/−^ mice, where *P* is <0.05 (Student’s *t* test).

We first examined the relative cytokine and chemokine levels in serum samples collected 6 days following EBOV challenge by using a multiplex-based bead assay. Quantitative analysis revealed that multiple Th1 cytokines, including gamma interferon (IFN-γ), interleukin-2 (IL-2), and tumor necrosis factor alpha (TNF-α), were significantly reduced in Tim-1^−/−^ mice compared to EBOV-infected wild-type mice (Fig. 2A to C), while IL-12p40 was increased (Fig. 2B). Similarly, the Th2 cytokines IL-4 and IL-10 were also significantly reduced (Fig. 2C). We also detected a significant reduction of granulocyte-macrophage colony-stimulating factor (GM-CSF) (see Table S1 in the supplemental material), which stimulates stem cells to differentiate into granulocytes and monocytes (46). Following activation, prolonged Tim-1 expression was shown on both Th1 (~1 week) and Th2 (~3 weeks) cells (47); hence, the decrease in production of most of the Th1 and Th2 cytokines may be due to the absence of the costimulatory function of Tim-1, which normally occurs following TCR engagement with antigen-presenting cells (APCs) through upregulation of a Tim-1 ligand, Tim-4 (reviewed in reference 48). Flow cytometry analysis demonstrated that at day 6, gated CD4^+^ T cells from Tim-1^−/−^ mice appeared to produce elevated levels of IL-2, IFN-γ, and TNF-α compared to wild-type, EBOV-infected mice (Fig. 2D). Unexpectedly, analysis of viremia indicated that Tim-1^−/−^ mice had nearly equivalent levels of viral genomes per milliliter of serum (Fig. 2E). These findings suggest that (i) Tim-1 may be one of several receptors that support viral attachment and/or entry and (ii) despite high plasma viremia, the decrease of inflammatory mediators or the development of an altered immunological response may have led to survival of Tim-1^−/−^ mice. Taken together, our data suggest that Tim-1 is critically involved in EVD; however, Tim-1^−/−^ mice exhibited clinical signs associated with EVD during the early phase of disease despite the significant increase in survival. Furthermore, a robust, albeit altered, inflammatory response and the presence of nearly equivalent viremia levels in Tim-1^−/−^ mice in comparison to WT mice are consistent with the presence of additional attachment factors for EBOV. Previous studies have identified the lectins DC-SIGN and L-SIGN (49–51), folate receptor α (52), and Tyro3 receptor tyrosine kinases (53) as attachment factors for the virus.

10.1128/mBio.00845-17.9 TABLE S1  Analysis of cytokines and chemokines in sera of wild-type and Tim-1^−/−^ mice infected with EBOV. Download TABLE S1, DOCX file, 0.1 MB. Copyright © 2017 Younan et al. 2017 Younan et al. This content is distributed under the terms of the Creative Commons Attribution 4.0 International license.

### EBOV directly binds to T lymphocytes, inducing a “superagonist-like” effect.

**FIG 3:**
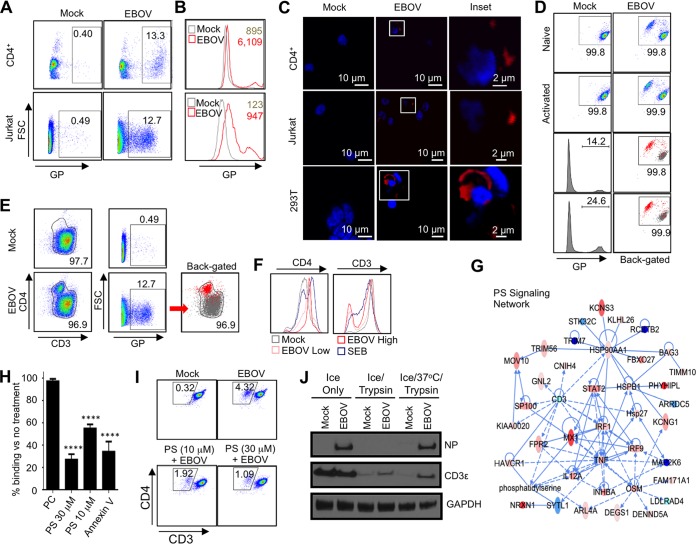
EBOV binds to CD4^+^ T cells and induces rapid TCR internalization in a PS-dependent manner. (A) EBOV binding to T cells. Isolated CD4^+^ T lymphocytes and Jurkat cells were incubated with EBOV for 2 h at 4°C, stained for GP, and analyzed by flow cytometry. FSC, forward scatter. (B) EBOV binding to CD4^+^ T lymphocytes (upper panel) and Jurkat cells (lower panel) demonstrating the GP^Hi^ population and the shift of the bulk population with mean fluorescence intensity indicated. (C) Confocal microscopy of EBOV bound to primary CD4^+^ T lymphocytes, Jurkat cells, and 293T cells. Insets show the formation of plasma membrane-associated GP-positive puncta. (D and E) Rapid induction of a CD4^Hi^ CD3^Low^ population by EBOV following a 2-h incubation at 4°C. Primary naive (upper panel) and activated (lower panel) CD4^+^ T lymphocytes (D) and Jurkat cells (E). The EBOV-GP^+^ populations were back-gated onto the CD4-versus-CD3 plots. (F) CD4 and CD3 expression levels 24 h after stimulation of CD4^+^ T cells with SEB or EBOV. (G) Activation of PS signaling, including significant upregulation of Tim-1 (HAVCR1), in isolated primary CD4^+^ T cells incubated with EBOV. Deep-sequencing-based transcriptional profiling in which solid lines represent direct interactions and dashed lines represent indirect interactions from IPA’s Knowledge Base. Red and blue indicate increased and decreased transcriptional activity, respectively. (H) Preincubation of CD4^+^ T cells with PS-containing liposomes or preincubation of EBOV with annexin V reduces viral binding to cells. ****, *P* < 0.0001 (Student *t*-test). (I) Addition of PS-containing liposomes reduces EBOV-induced development of CD4^Hi^ CD3^Low^ population. (J) Following EBOV binding, CD4^+^ T cells were either immediately treated with trypsin or incubated for 1 h at 37°C followed by trypsin treatment. EBOV binding and/or internalization was assessed by Western blotting for NP. In addition, blotting for CD3ε was used to determine the relative internalization of TCR. Binding assays are representative of over a dozen independent experiments; histograms are representative of one donor. Inhibition assays are representative of one of four individual experiments conducted in triplicate.

As noted, despite the ability of EBOV to lead to lymphopenia, the virus does not infect lymphocytes. Due to the involvement of Tim-1 signaling in the stimulatory effects on CD4^+^ T cells, we next sought to investigate if EBOV directly interacts with T cells. Flow cytometry binding assays were developed and validated using Vero-E6 and 293T cells, both of which are susceptible to EBOV infection. Cells were incubated with EBOV-green fluorescent protein (GFP) for 2 h at 4°C, washed, stained with anti-EBOV serum, and analyzed by flow cytometry (Fig. S1A). A high percentage of EBOV binding was observed with Vero-E6 cells (>60%), while 293T cells exhibited a reduced binding of ~16%. Unexpectedly, EBOV binding was similarly observed on primary CD4^+^ T cells and Jurkat cells (Fig. 3A). When cells were exposed to EBOV at a multiplicity of infection (MOI) of 1 PFU/cell, a shift in the bulk population of cells and the development of an EBOV glycoprotein (GP)^Hi^ population were detected in both CD4^+^ T cells (13.3%) and Jurkat cells (12.7%) (Fig. 3B). The MOI of 3 PFU/cell resulted in an increase of the GP^Hi^ population to 34% (Fig. S1B), suggesting that the effect is dose dependent. Confocal microscopy was then performed to determine the relative distribution of cell-bound EBOV at the plasma membrane. This analysis further confirmed a direct binding of EBOV particles to purified human CD4^+^ T cells and Jurkat cells, as well as 293T cells (Fig. 3C). We noted that the short 2-h-long incubation of primary CD4^+^ T lymphocytes with EBOV resulted in a rapid development of a CD4^Hi^ CD3^Low^ population in isolated primary CD4^+^ T cells and Jurkat cells (Fig. 3D and E). Overnight CD3/CD28 bead activation followed by 2-h GP binding analysis resulted in an increase in the CD4^Hi^ CD3^Low^ population of EBOV-positive cells compared to nonstimulated cells (Fig. 3D, lower panel). Back-gating of GP-bound cells revealed that the vast majority of binding occurred in the CD4^Hi^ CD3^Low^ population (Fig. 3D and E, back-gated). A rapid and specific downregulation of CD3 is a major characteristic feature of the effect of staphylococcal enterotoxin B (SEB) superantigen (54); we therefore compared the effects of EBOV and SEB on the expression of CD4 and CD3. Following 4 days of culture, a striking similarity between the effects of EBOV and SEB was observed, the former being clearly dose dependent (Fig. 3F).

10.1128/mBio.00845-17.1 FIG S1  EBOV binding and internalization in permissive and nonpermissive cell types. (A) Binding of EBOV to Vero-E6 and 293T cells, both of which are susceptible to EBOV infection, following a 2-h incubation at 4°C. Staining was performed as described for Fig. 1. Representative data from two independent experiments. (B) Binding of EBOV to isolated CD4^+^ T cells at the indicated MOIs (PFU/cell). Data representative of 3 donors. Both panels show flow cytometry data with percentages of the gated GP^+^ populations. (C) Western blot demonstrating reduced full-length CD3ζ and increased CD3ζ cleavage product in EBOV-stimulated samples. (D) Confocal microscopy demonstrating intracellular colocalization of EBOV and CD3ε within Rab7^+^ late endosomes following exposure of CD4^+^ T cells to EBOV in the presence of chloroquine. Representative data from one of three independent donors. Download FIG S1, PDF file, 0.3 MB. Copyright © 2017 Younan et al. 2017 Younan et al. This content is distributed under the terms of the Creative Commons Attribution 4.0 International license.

Recent findings demonstrated that virion-associated phosphatidylserine (PS) is sufficient to permit EBOV binding to cells susceptible to the virus, which is followed by internalization of virions attached to PS-binding receptors (40, 42). We therefore sought to determine if binding of EBOV to CD4^+^ T cells triggers PS-associated signaling pathways. Indeed, transcriptome analysis demonstrated that EBOV stimulation induced PS-related signaling as early as 24 h postinfection, the earliest time point analyzed, and Tim-1 (HAVCR1) was among the significantly differentially expressed genes (Fig. 3G). We therefore examined whether EBOV binding to T lymphocytes could be inhibited through the addition of PS-containing liposomes to cell cultures. Indeed, when PS-liposomes were added to purified CD4^+^ T cells 1 h prior to the addition of EBOV, viral binding was reduced in a dose-dependent manner, whereas no reduction was observed following the addition of phosphatidylcholine (PC)-containing vesicles (Fig. 3H). PS is present on the EBOV membrane (55); the addition of annexin V, a PS-binding substrate, to virions prior to addition to T cells also reduced EBOV binding. Consistent with the decreased binding, the addition of PS-containing liposomes significantly reduced the development of the CD4^Hi^ CD3^Low^ population observed in our binding assay (Fig. 3I).

Next, we attempted to determine if EBOV internalized together with the TCR complex. CD4^+^ T cells were cultured with EBOV for 2 h at 4°C to promote binding, washed, and treated with trypsin either immediately or following a 1-h incubation at 37°C. Western blot analysis demonstrated that EBOV NP protein was detectable only in control nontreated cells and samples cultured at 37°C prior to trypsin digestion, suggesting that the viral particles were internalized (Fig. 3J). Consistent with our flow cytometry analysis, the CD3ε subunit of the TCR complex was higher in EBOV-treated samples, further providing evidence of rapid TCR internalization. Similarly, EBOV stimulation of naive and CD3/CD28-activated CD4^+^ T cells resulted in the reduction of full-length CD3ζ, with an increase in cleavage product of CD3ζ being detected in EBOV-stimulated samples (Fig. S1C). Taken together, these results indicate that the TCR complex is internalized and degraded rapidly following exposure to EBOV. Confocal microscopy experiments further demonstrated colocalization of EBOV and CD3ε within Rab7^+^ late endosomes (Fig. S1D). Despite EBOV internalization, no viral replication was detected in T cells (Fig. S2). Consistent with these results, comparative transcriptome analysis indicated that EBOV stimulation of CD4^+^ T cells induced a significant upregulation of genes whose interactions together enrich for functional pathways known to be induced by superagonists (Fig. S3). Significantly upregulated genes in this pathway, including IRF1, IRF7, and ZBP1, have been previously described as participants in global host transcriptional responses to superagonist (56).

10.1128/mBio.00845-17.2 FIG S2  EBOV does not infect T lymphocytes. Isolated CD4^+^ T lymphocytes and CD8^+^ T lymphocytes were activated with CD3/CD28 beads or kept nonactivated; incubated with EBOV-GFP at an MOI of 2 PFU/cell for 1, 4, or 7 days; and analyzed by flow cytometry. Infection of dendritic cells (DC) was used as a positive control. The data are representative of 3 independent donors. Download FIG S2, PDF file, 0.2 MB. Copyright © 2017 Younan et al. 2017 Younan et al. This content is distributed under the terms of the Creative Commons Attribution 4.0 International license.

10.1128/mBio.00845-17.3 FIG S3  EBOV triggers response similar to known superagonists. Transcriptome analysis of EBOV-exposed versus mock-treated CD4^+^ T cells on day 1. Genes upregulated due to EBOV treatment are shown in red. Network is enriched for interactions related to cell signaling that are characteristic of a superantigen response. Solid lines represent direct interactions, and dotted lines represent indirect interactions from IPA’s Knowledge Base. Download FIG S3, PDF file, 0.1 MB. Copyright © 2017 Younan et al. 2017 Younan et al. This content is distributed under the terms of the Creative Commons Attribution 4.0 International license.

### Binding of EBOV induces T-cell activation and cytokine production.

**FIG 4:**
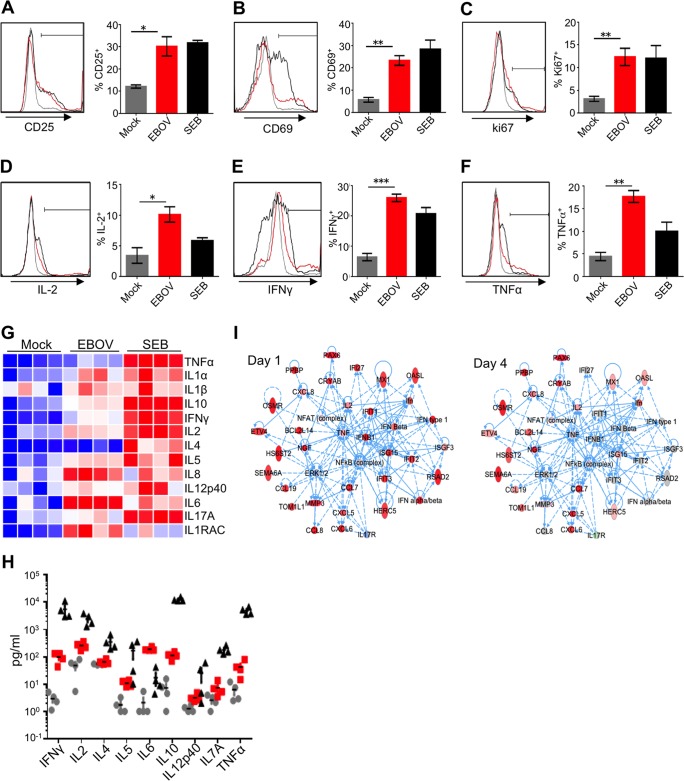
EBOV activates CD4^+^ T cells and induces the release of inflammatory mediators. (A to C) Expression levels of the activation markers CD25 (A), CD69 (B), and intracellular Ki-67 (C) as assessed by flow cytometry at 48 h after addition of EBOV to CD4^+^ T cells. (D to F) Intracellular cytokine staining for IL-2 (D), IFN-γ (E), and TNF-α (F) performed on primary CD4^+^ T cells at 48 h after the addition of EBOV at an MOI of 0.3; histograms of cells from representative donors and mean percentages of cells from 4 donors ± SE. Statistical significances between the mean percentages are shown as follows: *, *P* < 0.05; **, *P* < 0.01; ***, *P* < 0.001 (Student’s *t* test). (G and H) Heat maps and levels of cytokines (picograms per milliliter) associated with Th1, Th2, and Th17 responses in supernatants of EBOV-stimulated CD4^+^ T cells at 48 h after addition of EBOV. Heat maps in panel G are shaded as in Fig. 2A. Grey circles, red squares, and black triangles represent mock- (medium only), EBOV-, and SEB-stimulated cells, respectively. (I) Transcriptional profiling shows the coordinated upregulation in inflammation pathways indicative of a cytokine storm in CD4^+^ T cells on both day 1 and day 4 after addition of EBOV. Solid lines represent direct interactions, and dashed lines represent indirect interactions from IPA’s Knowledge Base. Expression data from EBOV-infected samples relative to mock samples at day 1 and day 4 are overlaid onto each gene, where red represents significant relative upregulation and blue represents significant relative downregulation.

We next determined the relative extent of EBOV-induced activation of T cells by comparing populations positive for markers of activation and proliferation in peripheral blood mononuclear cells (PBMCs), PBMCs depleted of target cells (DCs and monocytes), and isolated CD4^+^ T cells (Fig. 4). A 48-h-long incubation of these cells in the presence of EBOV resulted in upregulation of the percentages of CD25^+^, CD69^+^, and Ki-67^+^ populations compared to cells incubated without the virus. Typically, addition of EBOV to PBMC cultures resulted in less than a 2-fold increase in the percentages of activated CD4^+^ T cells, and when DCs and monocytes were depleted, a 2- to 2.5-fold increase was detected (Fig. S4). However, the addition of EBOV to isolated CD4^+^ T cells resulted in the highest relative increase of populations positive for all three activation markers, with a 3.5- to 4.5-fold increase being observed (Fig. 4A to C). These findings indicate that CD4^+^ T cells are activated more efficiently in the absence of known target cells, demonstrating that EBOV directly stimulates CD4^+^ T cells.

10.1128/mBio.00845-17.4 FIG S4  EBOV binding induces activation of T lymphocytes in PBMCs and PBMCs devoid of monocytes and DCs. Expression levels of the indicated activation markers on CD4^+^ T cells assessed by flow cytometry at 48 h after addition of EBOV to total PBMCs (A and C) or PBMCs in which DCs and monocytes had been depleted (D to F). Mean values ± SE from 4 donors. *, *P* < 0.05; **, *P* < 0.01 (Student’s *t* test). Download FIG S4, PDF file, 0.3 MB. Copyright © 2017 Younan et al. 2017 Younan et al. This content is distributed under the terms of the Creative Commons Attribution 4.0 International license.

As noted, we observed a striking similarity between stimulation of CD4^+^ T cells by EBOV and known superagonists. A characteristic feature of superagonist activity is the release of inflammatory mediators. Nuclear factor of activated T cells (NFAT) is the key transcriptional regulator of inflammatory mediators (57). We therefore examined the phosphorylation kinetics of NFAT-1 following incubation of CD4^+^ T cells and Jurkat cells with EBOV. We found that EBOV stimulation results in the induction of the NFAT signaling pathway, as evidenced by a rapid induction of the activated, monophosphorylated form of NFAT-1; a similar effect was detected following addition of known inducers of NFAT, 12-*O*-tetradecanoylphorbol-13-acetate (TPA)–ionomycin or staphylococcal enterotoxin B (SEB) (Fig. S5A and B). Consistent with these findings, EBOV stimulation of Jurkat cells transfected with a construct expressing luciferase under the control of an NFAT promoter resulted in increased luciferase activity (Fig. S5C). In order to directly assess the resulting activation of T cells contributing to inflammatory response, CD4^+^ T cells were incubated in the presence of EBOV for 48 h and analyzed by flow cytometry. The analysis demonstrated the increased percentages of cells positive for IL-2, IFN-γ, and TNF-α compared to cells incubated without the virus (Fig. 4D to F).

10.1128/mBio.00845-17.5 FIG S5  EBOV induces NFAT activity in CD4^+^ T cells. (A and B) Phosphorylation of NFAT-1 in primary CD4^+^ T cells (A) and Jurkat cells (B) stimulated with 25 ng/ml TPA and 0.5 μM ionomycin, with or without 10 μM CsA, 1 μg/ml SEB, or EBOV at an MOI of 5 PFU/cell. Representative data from three independent experiments performed with different donors. GAPDH was used to normalize sample loading. (C) Jurkat cells were transfected with an NFAT-dependent luciferase reporter construct; incubated for 24 h; and stimulated for 6 h with medium (mock), EBOV at an MOI of 5, or TPA/ionomycin; and lysed. Average luciferase activity shown for triplicate wells ± SE. Download FIG S5, PDF file, 0.2 MB. Copyright © 2017 Younan et al. 2017 Younan et al. This content is distributed under the terms of the Creative Commons Attribution 4.0 International license.

Analysis of supernatants of EBOV-infected PBMCs using a bead-based multiplex assay demonstrated the increased production with both Th1 and Th2 cytokines and CXCL8 chemokine (IL-8), also produced by T cells (58), compared to uninfected PBMCs. The effect was strongly increased when macrophages and DCs were depleted and was maximal with purified CD4^+^ T cells (Fig. 4G and H and S6A and B and Table S2). This is consistent with the greater fold increase in activation markers detected in isolated CD4^+^ T cells in comparison to EBOV-infected total PBMCs and PBMCs with DCs and macrophages depleted (Fig. 4A to C and S4). Analysis of cells after stimulation with SEB demonstrated greater concentrations of cytokines in supernatants; however, intracellular cytokine levels were lower as determined by flow cytometry (Fig. 4D to F and S6C to H), which suggests a more rapid kinetics of activation. Comparative transcriptome analysis of previously identified pathways at days 1 and 4 after stimulation of CD4^+^ T cells with EBOV indicated that the response is consistent with the coordinated upregulation of genes characteristic of a cytokine storm (Fig. 4I). While the response in this inflammation network was much more pronounced on day 1, many of these genes still remained significantly upregulated on day 4.

10.1128/mBio.00845-17.6 FIG S6  EBOV-mediated induction of cytokines in PBMCs or PBMCs devoid of monocytes, DCs, and T cells. Heat maps and levels of cytokines (pg/ml) associated with Th1, Th2, and Th17 responses in supernatants of EBOV-stimulated PBMCs (A) or no-target PBMCs (with macrophages and DCs depleted) (B) at 48 h after addition of EBOV. Data from 4 individual donors; see Table S2 for statistical comparisons. (C to H) Intracellular cytokine staining performed 48 h after stimulation of CD4^+^ T cells by EBOV at an MOI of 1 PFU/cell: IL-2 (C and F), IFN-γ (D and G), and TNF-α (E and H) in total PBMCs (C to E) and PBMCs with monocytes and DCs depleted (F to H). Histograms are representative primary flow cytometry data of one out of four donors. Average values ± SE based on results from 4 donors. *, *P* < 0.05 (Student’s *t* test). Download FIG S6, PDF file, 0.3 MB. Copyright © 2017 Younan et al. 2017 Younan et al. This content is distributed under the terms of the Creative Commons Attribution 4.0 International license.

10.1128/mBio.00845-17.10 TABLE S2  Cytokine response of immune cells to EBOV. Download TABLE S2, DOCX file, 0.1 MB. Copyright © 2017 Younan et al. 2017 Younan et al. This content is distributed under the terms of the Creative Commons Attribution 4.0 International license.

### EBOV binding and activation of primary CD4^+^ T cells is associated with Tim-1.

**FIG 5:**
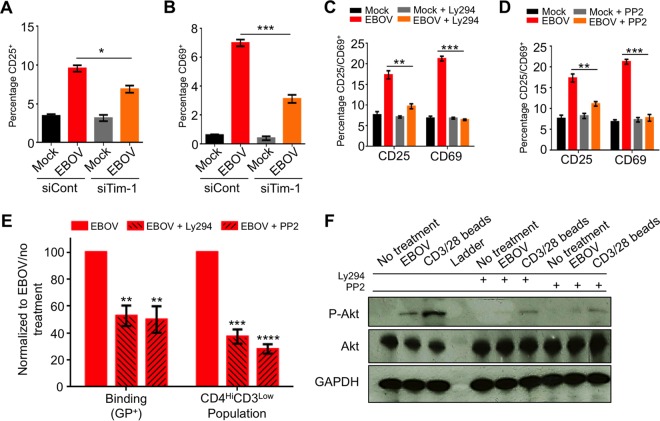
Role of Tim-1 in EBOV-induced activation of CD4^+^ T cells. (A and B) Disabling of Tim-1 reduces activation of EBOV-exposed T cells. Jurkat cells were transfected with control siRNA or siRNA targeting Tim-1, incubated for 48 h, stimulated with EBOV for an additional 48 h, stained, and analyzed for CD25 (A) and CD69 (B). (C) The EBOV-mediated activation of T cells depends on Lck kinase. Primary CD4^+^ T cells were incubated with Src inhibitor Ly294 for 1 h, stimulated with EBOV for 48 h, and analyzed for CD25 and CD69. (D) The EBOV-mediated activation of T cells depends on the PI3K pathway. Primary CD4^+^ T cells were incubated with PI3K inhibitor PP2 and EBOV and analyzed as described for panel F. (E) Binding of EBOV GP and EBOV-mediated downregulation of CD3 depend on Lck kinase and PI3K. Primary CD4^+^ T cells were incubated with Ly294 or PP2, and the percentages of binding and the CD4^Hi^ CD3^Low^ population were determined. (F) EBOV-induced phosphorylation of Akt depends on Src kinase and PI3K. CD4^+^ T cells were treated with Ly294 and PP2. Akt and its phosphorylated form were analyzed by Western blotting. *, *P* < 0.05; **, *P* < 0.01; ***, *P* < 0.001; ****, *P* < 0.0001 (Student *t*-test).

Previous reports indicated that Tim-1 signaling is sufficient to activate primary CD4^+^ T cells, and under certain conditions, Tim-1 may act as a costimulator of T cells, leading to increased activity (59). We therefore analyzed the role of Tim-1 in activation of primary CD4^+^ T cells exposed to EBOV. Targeting of Tim-1 by small interfering RNA (siRNA) significantly reduced activation of T cells, based on expression of CD25 and CD69 (Fig. 5A and B). Signal transduction through Tim-1 was shown to result in Lck (Src kinase family)-dependent phosphorylation of the cytoplasmic tail of Tim-1, resulting in activation of the phosphatidylinositol 3-kinase (PI3K) pathway (59). Consistent with the role of Lck signaling, exposure of the Jurkat-derived cell line deficient for Lck, J.Cam1.6, to EBOV did not result in the downregulation of plasma membrane-associated CD3 or the upregulation of CD25 or CD69 and showed almost no binding of GP (Fig. S7A to D). Moreover, the addition of the Src kinase inhibitor Ly294 or the PI3K inhibitor PP2 significantly reduced the expression of both CD25 and CD69 (Fig. 5C and D). Unexpectedly, treatment with Ly294 or PP2 also resulted in a decrease in EBOV binding, which coincided with a significant drop in the development of the previously observed CD4^Hi^ CD3^Low^ population (Fig. 5E) and reduction in Tim-1 expression (Fig. S7E and F). Therefore, the reduction of both EBOV binding and the CD4^Hi^ CD3^Low^ population is likely associated with the reduced Tim-1 expression. The ability of Lck and PI3K inhibitors to block cellular activation was further verified with primary CD4^+^ T cells and Jurkat cells following stimulation with CD3/CD28 beads in the presence or absence of the inhibitors (Fig. S8A to D). Moreover, expression of Tim-1 in J.Cam1.6 cells was lower than in Jurkat cells, suggesting that Tim-1 expression may be associated with basal Lck activity during a nonproliferative state. Further demonstrating the role of the PI3K pathway in EBOV-induced activation, both PP2 and Ly294 blocked EBOV-induced activation and phosphorylation of Akt, which is dependent upon PI3K activity (60) (Fig. 5F and S8E to H). These findings demonstrate that binding of EBOV to Tim-1 on the surface of primary T lymphocytes results in their direct activation, suggesting a direct role of Tim-1 in the development of a cytokine storm and pathogenesis associated with EBOV-infection.

10.1128/mBio.00845-17.7 FIG S7  EBOV stimulation is Lck pathway dependent. (A to D) The T-cell downregulation of CD3 and activation depend on Lck kinase. (A) Surface expression of CD3. (B to D) Binding of GP (B), CD25 (C), and CD69 (D) in Lck-deficient Jurkat-derived cell line J.Cam1.6 incubated with EBOV for 48 h. GP binding was determined on J.Cam1.6 cells using the previously described binding assay. (E and F) Jurkat cells pretreated for 1 h with an inhibitor of Src kinase, Ly294 (10 μM) (E), or an inhibitor of PI3K, PP2 (10 μM) (F), and cultured for 3 days in the presence of EBOV at an MOI of 1 PFU/cell. Expression of Tim-1 analyzed by flow cytometry. Percentages of Tim-1^+^ cells normalized to untreated cells. Mean of triplicate samples ± SE of the mean from one of two donors. Download FIG S7, PDF file, 0.1 MB. Copyright © 2017 Younan et al. 2017 Younan et al. This content is distributed under the terms of the Creative Commons Attribution 4.0 International license.

10.1128/mBio.00845-17.8 FIG S8  Inhibition of Src kinase or PI3K reduces activation of primary CD4^+^ T cells and Jurkat cells. Primary CD4^+^ T cells were pretreated for 1 h with an inhibitor of Src kinase, Ly294, or an inhibitor of PI3K, PP2, and cultured for 3 days in the presence of anti-CD3/CD28-coated beads. (A to D) The percentages of inhibition were determined by analysis of expression of the cellular activation markers CD25 (A and C) and CD69 (B and D) by flow cytometry. (E to H) Jurkat cells were pretreated for 1 h with an inhibitor of Src kinase, Ly294, or an inhibitor of PI3K, PP2, and cultured for 3 days in the presence of EBOV at an MOI of 1 PFU/cell. The percentages of inhibition were determined by analysis of expression of the cellular activation markers CD25 (E and F) and CD69 (G and H) by flow cytometry. Mean values ± SE based on triplicates from one of two independent donors. *, *P* < 0.05 (Student’s *t* test). Download FIG S8, PDF file, 0.2 MB. Copyright © 2017 Younan et al. 2017 Younan et al. This content is distributed under the terms of the Creative Commons Attribution 4.0 International license.

### EBOV predominantly binds to T_CM_ cells and activates CD4^+^ T cells in the absence of target cells.

**FIG 6:**
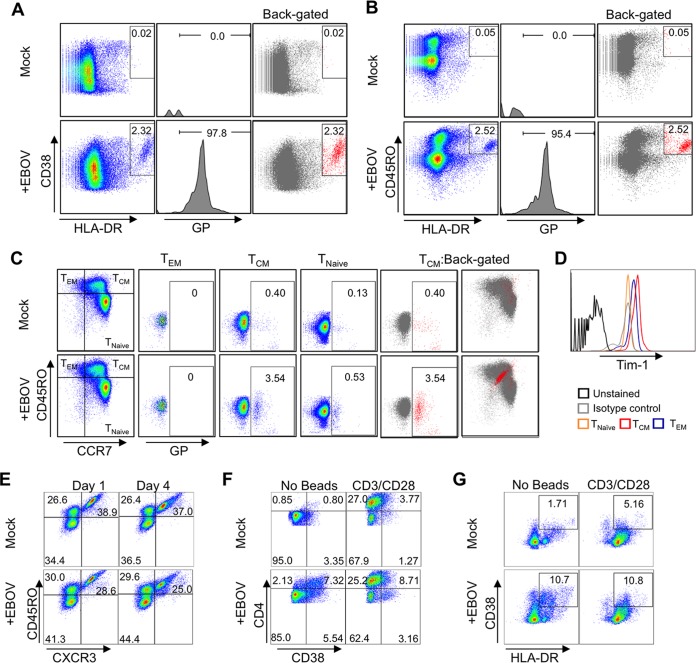
EBOV preferentially binds to T_CM_ cells. (A) Activation of isolated CD4^+^ T cells by EBOV. Expression of HLA-DR versus CD38 (left panel). The EBOV GP^+^ population (middle panel) was back-gated on HLA-DR^+^ CD38^+^ flow cytometry plots (right panel) to determine correlation of GP binding with cell activation. (B) HLA-DR versus CD45RO staining used to determine if activated cells are derived from the naive (CD45RO^−^) or memory (CD45RO^+^) subsets. GP^+^ population (middle panel) was back-gated to HLA-DR^+^ CD45RO^+^ plots (right panel). (C) EBOV binding to T_EM_ or T_CM_ determined following staining for CCR7 versus CD45RO. GP binding for each of the 3 quadrants corresponding to T_EM_, T_CM_, and naive cells is shown. GP^+^ cells were back-gated to CCR7^+^ CD45RO^+^ plots for T_CM_ quadrant (right panel). (D) Tim-1 expression profile on T-cell subsets. (E) Analysis of Th subsets in primary CD4^+^ T cells following 1- and 4-day-long stimulation with EBOV. Percentages of naive (CD45RO^−^), Th2/17 (CD45RO^+^ CXCR3^−^), and Th1 (CD45RO^+^ CXCR3^+^) cell populations. (F) Analysis of CD4^Hi^ CD38^+^ T cells after 4-day-long stimulation with EBOV, CD3/CD28 beads, or both EBOV and CD3/CD28 beads. (G) Analysis of HLA-DR^+^ CD38^+^ T cells at 4 days following stimulation with EBOV, CD3/CD28 beads, or both EBOV and CD3/CD28 beads. Data in all panels are representative of 1 of 2 independent donors and experiments performed in triplicate wells.

We next sought to determine whether EBOV binding was random or if specific CD4^+^ T-cell subsets were targeted. A reduced MOI of 0.3 PFU/cell was used to prevent saturation of binding; otherwise, the binding was performed as previously described. As indicated in Fig. 6A (left panel), the addition of EBOV induced the development of a distinct population staining double positive for HLA-DR and CD38, which represents an activated CD4^+^ T-cell subset. The GP^+^ population (middle panel) was then back-gated onto the HLA-DR-versus-CD38 plots (right panel). A clear overlap between the GP^+^ population and the activated subset was apparent, further indicating that EBOV directly activates primary human CD4^+^ T cells. To determine if EBOV preferentially bound and activated naive or memory cells, cells were stained with CD45RO and HLA-DR (Fig. 6B). The majority of GP^+^ cells staining positive for HLA-DR costained for CD45RO, indicating that EBOV binding was associated with CD4^+^ memory T cells. We then assessed whether EBOV bound to a specific subset of memory cells following costaining with CD45RO and CCR7 (Fig. 6C). GP binding was assessed in the three quadrants representing CD45RO^+^ CCR7^−^ effector memory T cells (T_EM_), CD45RO^+^ CCR7^−^ central memory cells (T_CM_), and CD45RO^−^ CCR7^−^ naive cells (T_Naïve_). A GP^+^ population was primarily observed in T_CM_ cells cultured in the presence of EBOV; back-gating to CCR7-versus-CD45RO plots revealed a distinct population within the central memory quadrant. These findings indicate that EBOV preferentially binds to a specific subset of T_CM_ cells. Further investigation is under way to determine the precise phenotype and identity of this subset of EBOV-binding T cells; however, as previously reported, Tim-1 expression was highest on T_CM_ cells (Fig. 6D).

We next examined the effects of short-term (1- and 4-day) cultures of isolated CD4^+^ T cells in the presence of EBOV (MOI of 0.3 PFU/cell) on the relative percentages of Th2/Th17 cells versus Th1 cells by flow cytometry. A decrease in the percentages of the Th1 (CD45RO^+^ CXCR3^+^) cells was consistently observed at both time points (Fig. 6E). This finding suggests that EBOV may preferentially reduce the Th1 cells or lead to expansion of the Th2/Th17 cells. During our analysis, we noted an increase in CD4 expression, which is consistent with cellular activation and division of stimulated cells (61). The majority of CD4^Hi^ cells costained for the activation marker CD38 (Fig. 6F, bottom left panel). Of note, the addition of EBOV to cells prestimulated with CD3/CD28 activation beads consistently resulted in higher levels of activation than that for cells stimulated with CD3/CD28 beads alone (Fig. 6F, top right versus bottom right). As expected, stimulation with CD3/CD28 beads further upregulated the CD4 receptor on EBOV-exposed T cells. Further analysis revealed a greater increase in the percentages of HLA-DR^+^ CD38^+^ T cells when cells were costimulated with activation beads and EBOV than when stimulated with beads alone (Fig. 6G). Overall, these findings indicate that EBOV preferentially binds and activates a subset within the T_CM_ population and also further stimulates CD4^+^ T cells following TCR-mediated activation.

## DISCUSSION

Our *in vivo* studies demonstrated the central role of Tim-1 in EVD pathogenesis and indicate a putative therapeutic intervention strategy. Previous *in vivo* studies have demonstrated that simultaneous infusion of an agonistic Tim-1-specific antibody with antigen results in increased IL-4- and IFN-γ-expressing CD4^+^ T cells and prevents the development of respiratory tolerance and increased pulmonary inflammation (47). We note that while both IL-4 and IFN-γ were significantly elevated in wild-type EBOV-infected mice, a statistically significant decrease in both cytokines was detected in serum of EBOV-infected Tim-1^−/−^ mice. Similarly, another study demonstrated that administration of a Tim-1 natural ligand, Tim-4–Ig fusion protein, resulted in hyperproliferation of T cells (62). In the course of EVD, it is likely that EBOV acts as both an antigen (e.g., following infection of APCs and viral peptide presentation on major histocompatibility complexes [MHCs]) and a Tim-1 activator through interactions with virion-associated PS. In this regard, EBOV fulfills the requirement for both the initial MHC-TCR-dependent activation of T cells and a secondary, costimulating signal through virion-associated engagement with Tim-1, which may lead to the hyperactivation observed in recent human cases (63) and contribute to the cytokine storm following EBOV infection.

As evidenced in these studies, however, the combination of our cytokine/chemokine analyses, flow cytometry data, transcriptome profile, and the observed rapid internalization of CD3 suggests that EBOV possesses a “superagonist-like” activity, as activation is observed in the absence of detectable viral infection/replication in CD4^+^ T cells or the Jurkat cell line, indicating TCR-independent activation. We note that this is consistent with relative effects of anti-Tim-1 antibodies (59), which showed TCR-independent activation. These findings have demonstrated that Tim-1 signaling can directly activate T cells without concomitant TCR-dependent costimulation. Tim-4, a ligand for Tim-1, has been shown to induce CD4^+^ T-cell expansion and phosphorylation of Akt and extracellular signal-regulated kinases 1 and 2 (ERK1/2) while promoting release of both Th1- and Th2-type cytokines, which is consistent with our findings (64, 65). In addition, a study by Mariat et al. demonstrated that antibody stimulation of Tim-1 induces CD4^+^ T-cell proliferation in the absence of MHC class II-dependent signals both *in vitro* and *in vivo* (66). Interestingly, and in support of our findings, the study also indicated that Tim-1 stimulation also induced NFAT signaling and cytokine production (66). Furthermore, de Souza et al. demonstrated that anti-Tim-1 antibodies induce CD69 and CD25 expression on naive murine CD4^+^ T cells in the absence of TCR stimulus (59). As shown in our study, siRNA targeting of Tim-1 reduced both CD69 and CD25 (Fig. 5A and B). Notably, non-TCR-dependent activation of CD4^+^ T cells has been previously reported, as anti-CD28 antibodies alone are sufficient to induce mass proliferation of T cells (67). Interestingly, activation with anti-CD28 antibodies primarily induced activation of central memory CD4^+^ T cells, which, as indicated previously, are the subset to which EBOV preferentially binds in our *in vitro* binding assays (Fig. 3C).

As we noted, EBOV stimulation of CD4^+^ T cells appears to be dependent on both Tim-1 expression and Lck, as Tim-1–siRNA knockdown and pharmaceutical inhibitors reduced EBOV binding and activation. These results are consistent with the previous observations demonstrating that Tim-1 signaling events are Lck dependent (59). Within the TCR complex, Tim-1 is directly associated with CD3 (65); given this physical interaction, it is logical to assume that cross-linking of Tim-1 would have similar effects as TCR engagement (66). The rapid internalization of the CD3 complex, as observed by flow cytometry and trypsin digestion assays, is consistent with TCR-complex downregulation following activation. Although previous studies have suggested that EBOV GP may shield MHC detection (68, 69), we note that these studies were performed in transduced and transfected DCs where GP is highly expressed; as CD4^+^ T cells are refractory to infection and a relatively low MOI of 1.0 was used for the majority of these studies, it is highly improbable that the decrease in CD3 expression is due to shielding effects.

We suggest that EBOV triggers Tim-1 signaling in a manner similar to anti-Tim-1 antibodies; however, it is likely that numerous PS–Tim-1 interactions between the long, filamentous EBOV virions and the surface of T cells result in localized cross-linking at the plasma membrane, which may further promote activation. Furthermore, the additive effect of culturing T cells in the presence of EBOV and CD3/CD28 beads is consistent with findings showing that anti-Tim-1 and CD3/CD28 costimulation results in a synergistic increase in activation (36). We note that although antibody stimulation of Tim-1 alone induces activation of T cells, it is feasible and perhaps likely that EBOV binding may induce secondary signals, which cumulatively result in an increased activation.

Tim-1 is upregulated on both activated Th1 and Th2 cells, although its extended expression following activation is normally observed in the Th2 cells (1 versus 3 weeks); importantly, several abovementioned studies, which utilized anti-Tim-1 antibodies to activate CD4^+^ T cells, demonstrated a basal level of Tim-1 expression by both primary CD4^+^ T cells and Jurkat cells (47, 59, 62). As observed in this study, activation of primary CD4^+^ T cells prior to incubation with EBOV resulted in increased binding (Fig. 3D), which is consistent with the increased surface expression of Tim-1 following T-cell activation (45). Furthermore, we show that the majority of EBOV binding was detected in the central memory subset, which is consistent with the elevated Tim-1 expression levels on this specific subset as shown in Fig. 6D and as previously observed in murine memory cells (47). It is important to note that other cell types expressing Tim-1, including DCs, monocytes, B cells, and epithelial cells (48, 70), may contribute to the onset of a cytokine storm following PS-dependent activation. Thus, our data indicate a putative role of PS-dependent signaling in the development of a cytokine storm through direct interaction with virion-associated PS. Additionally, the high levels of circulating apoptotic bodies and released cellular components exposing surface PS may further contribute to the onset of a cytokine storm by further stimulating the PS-dependent signaling pathways in CD4^+^ T cells and other cell types.

The findings presented here demonstrate for the first time that despite the rapid loss and limited cytokine secretion by EBOV-infected monocytes and DCs, EBOV directly binds to and activates CD4^+^ T cells in a TCR-independent manner, leading to the release of inflammatory mediators. We propose that the combination of nonspecific activation and increase of inflammatory mediators is among the primary contributing factors in the development of a cytokine storm. As our recent study demonstrates that EBOV glycoprotein directly triggers T-cell death (23), some of the cytokines associated with cell death, such as TNF-α, are likely to contribute to cytokine storm. These findings are supported in part by a recent report demonstrating that EBOV-infected patients exhibited relatively high levels of T-lymphocyte activation despite experimental therapies (63). As EBOV-infected DCs and monocytes fail to efficiently activate T cells (13), we suggest that EBOV directly activates T cells *in vivo*. Since the extent of the inflammatory response to EBOV is highly correlative with severity of disease and fatal outcomes (21, 71, 72), identification of factors contributing to the onset of the cytokine storm phenomenon may enable the development of novel therapeutics. In this regard, our data suggest that interfering with EBOV binding and/or activation of Tim-1 signaling appears to be a viable therapeutic strategy. This possibility is further supported by previous findings that have shown that inhibiting Tim-1 signaling on CD4^+^ T cells can reduce proliferation and production of inflammatory mediators, which may reduce the deleterious effects associated with a cytokine storm (32, 37, 54, 55).

The complexity of a cytokine storm response, which is characterized by the production of a series of overlapping and often redundant pro- and anti-inflammatory mediators, has limited the development of therapeutic intervention strategies (24, 37). Hence, strategies aimed at dampening the global inflammatory response to EBOV by limiting activation of the cells associated with cytokine production may provide a course of treatment. In this regard, tipping the scale in favor of maintaining immune homeostasis may be central to promoting recovery. Conversely, premature termination of an inflammatory response or therapeutically promoting an anti-inflammatory response following EBOV exposure may further contribute to “immune paralysis” and thereby lead to insufficient viremic control in recovering patients (24). A paradox exists between the induction of an aggressive immune response against EBOV and developing therapeutic strategies aimed at stemming the immune response, as survival or mortality requires an intricately balanced pro- and anti-inflammatory response.

Overall, our data suggest a putative role of PS sensing in the development of a cytokine storm response during the course of infection; however, based on our transcriptome analysis, it is feasible that EBOV directly stimulates additional, non-PS-dependent signaling pathways that lead to the production of cytokines following binding to T cells. The role of PS signaling in T lymphocytes is not well characterized. However, PS signaling has been shown to enhance TCR-dependent stimulation, leading to more robust activation (43). PS signaling is triggered by its ligands of the Tim family, which are expressed by many cell types (73). Engagement of T-cell-associated Tim-1 is linked to susceptibility to allergic and asthma responses. Conversely, Tim-3, which is primarily expressed by the Th1 cells, triggers mainly inhibitory signals that result in their apoptosis (reviewed in reference 73). Thus, virion-associated PS may trigger immune activation or tolerance, depending on the cell type and Tim expression profile.

Our data indicate that the phenotypic changes observed in CD4^+^ T cells stimulated with EBOV represent a novel, Tim-1-dependent signaling pathway triggered by EBOV that may contribute to the onset of a cytokine storm and activation-induced T-cell death (74), which may further contribute to the observed lymphopenia in EVD. Taken together, our results suggest that EBOV directly binds to and activates CD4^+^ T cells in a Tim-1-dependent manner. Although EBOV is readily internalized following PS-dependent binding, no productive viral replication occurs in T cells, suggesting the presence of a cellular restriction factor or the absence of a factor required for viral replication. These results also indicate that EBOV possesses “superagonist-like” activity due to increased proliferation in comparison to engagement following stimulation with anti-Tim-1 antibodies. It is feasible that EBOV binding to CD4^+^ T cells triggers additional, non-Tim-1-dependent signaling pathways, which may act as costimulators. Although disabling of Tim-1 dramatically increased survival (Fig. 1), disease was still observed, indicating that other pathways important for pathogenesis remain to be identified. Further studies are needed to determine the precise role of PS signaling *in vivo* and its putative role in the onset of a cytokine storm. Overall, these data contribute to understanding of the “immune paralysis” during EBOV infections.

## MATERIALS AND METHODS

### BSL-4 work.

All work with EBOV was performed within the Galveston National Laboratory biosafety level 4 (BSL-4) laboratories. For removal of mouse serum and cell-free supernatants of EBOV-infected cells from BSL-4, they were gamma irradiated using the 5-Mrad dose according to the approved University of Texas Medical Branch (UTMB) standard operating procedure protocol. Either flow cytometry was performed in BSL-4 using the Canto-II instrument (BD Biosciences), or cells were treated with 4% buffered paraformaldehyde in phosphate-buffered saline (PBS) for 48 h according to the approved UTMB standard operating procedure protocol and removed from BSL-4 for analysis with flow cytometers available in the UTMB Flow Cytometry Core Facility. Cell lysates for Western blot analysis were prepared by lysis of cells with 4× SDS-Laemmli buffer, followed by incubation at 95°C for 15 min, vortexing, and removal from BSL-4 laboratories. Cells for confocal microscopy were placed on slides; stained; fixed in 4% paraformaldehyde for 24 h, which was replaced with a fresh solution; incubated for an additional 48 h; and taken out of BSL-4. Staining and mounting procedures are described below.

### Mouse studies.

Infection of mice with mouse-adapted EBOV was approved by the University of Texas Medical Branch at Galveston Institutional Animal Care and Use Committee and performed in the BSL-4 containment facilities of the Galveston National Laboratory. Tim-1^−/−^ mice were kindly provided by Suzanne Cassel, University of Iowa, and described previously (75). Control C57BL/6J mice were purchased from Jackson Laboratories. Eight- to 9-week-old mice were infected by intraperitoneal injection with 1,000 PFU of mouse-adapted EBOV, strain Mayinga, which in a previous study was found to be equal to ~30,000 50% lethal doses (45). Mice were monitored twice daily from day 0 to day 14 postchallenge, followed by once-daily monitoring from day 15 to the end of the study at day 28. Clinical score was assessed as follows: (i) body weight, normal, 0; <10% loss, 1; 10 to 15% loss, 2; >20% loss, 3; (ii) appearance, normal, 0; lack of grooming, 1; rough coat, possible nasal and or ocular discharge, 2; very rough coat, abnormal posture, enlarged pupils, 3; (iii) clinical signs, normal, 0; small change of potential significance, 1; 25% rise in respiratory/heart rates, 2; 30% rise in respiratory/heart rates or markedly reduced/shallow respiratory/heart rates, paralysis, 3; (iv) unprovoked behavior, normal, 0; minor changes, 1; abnormal behavior, less mobile, less alert, inactive when activity expected, 2; unsolicited vocalization, extreme self-mutilation, 3; (v) behavior response to external stimuli, normal, 0; minor exaggerated response, 1; moderate abnormal response, 2. The overall score was tabulated and used to help interpret our assessment of each animal. A total score of 3 or less was considered normal; 4 to 7 indicated some evidence of pain or discomfort. Any animal reaching a total score of 8 or a score of 3 in any individual category was euthanized.

### Flow cytometry analysis of mouse lymphocytes.

Peripheral blood was collected in tubes containing EDTA (Kent Scientific Corp.; catalog identifier MTSC-EDTA) at day 6 postchallenge. Erythrocytes were lysed using lysis buffer (Sigma-Aldrich) as recommended by the manufacturer. Cells were pelleted at 400 × *g*, washed in phosphate-buffered saline containing 2% fetal bovine serum (Thermo Fisher Scientific), and stained with the following antibodies: CD3-brilliant UV 395 (BUV395) (145-2C11; BD Biosciences), CD4-peridinin chlorophyll protein (PerCP)/Cy5.5 (RM4-5; BD Biosciences), IFN-γ–phycoerythrin (PE) (XMG1.2; BD Biosciences), TNF-α–fluorescein isothiocyanate (FITC) (MP6-XT22; BD Biosciences), and IL-2–allophycocyanin (APC) (JES6-5H4; BD Biosciences).

### Multiplex analysis of serum cytokines and chemokine.

Serum samples were collected on day 6 post-EBOV infection. Samples were gamma irradiated with the 5-Mrad dose according to the UTMB standard operating procedure protocol, removed from the BSL-4 laboratory, and analyzed using a Multiplex-32 magnetic bead-based assay (Millipore) by Eve Technologies.

### Analysis of viremia.

Total RNA was isolated from serum samples taken at day 6 post-EBOV challenge using the QIAamp viral RNA minikit per the manufacturer’s protocol (Qiagen). EBOV was quantified with the one-step reverse transcription droplet digital PCR (RT-ddPCR) advanced kit for probes (Bio-Rad), with probes specific for the NP gene fragment corresponding to nucleotides 2095 to 2153 of EBOV genomic RNA (GenBank accession number AF086833) using forward primer GCCACTCACGGACAATGACA, reverse primer GCATGCGAGGGCTGGTT, and probe 6-carboxyfluorescein (FAM)–AGAAATGAACCCTCCGGCT-MGB. Briefly, 50 pg of RNA was added to 5 µl of supermix, 2 µl of reverse transcriptase enzyme, 1 µl of 300 mM dithiothreitol (DTT), and 1 µl of 20× NP custom TaqMan assay (Thermo Fisher Scientific) for each sample. ddPCR mixtures were loaded onto cartridges to create droplets on a QX200 droplet generator (Bio-Rad). The droplets were transferred onto 96-well PCR plates (Eppendorf) and amplified on a C1000 thermal cycler with a 96-deep-well reaction module (Bio-Rad). The following reaction conditions were used: 42°C for 60 min and 95°C for 10 min, followed by 39 cycles of 95°C for 15 s and 60°C for 1 min, and a final enzyme deactivation step of 98°C for 10 min. Finally, the PCR plates were loaded onto a droplet reader, which quantifies the number of positive and negative droplets in each sample. Analysis was performed using QuantaSoft software (Bio-Rad) to get the final concentrations in each sample.

### Viruses.

Recombinant EBOV, strain Mayinga, expressing GFP (76), was recovered from transfection of endotoxin-free plasmids harboring viral cDNA as previously described (11) and propagated by 3 passages in Vero-E6 cell monolayers. Viral stocks were quantified by plaque titration in Vero-E6 monolayers as previously described (77). Mouse-adapted EBOV, strain Mayinga (45), was obtained from the U.S. Army Medical Research Institute of Infectious Diseases (Frederick, MD), deposited at the World Reference Center of Emerging Viruses and Arboviruses (housed at UTMB), and amplified by a single passage in Vero-E6 cells.

### Cell lines.

Human embryonic kidney 293T (293T) and Vero-E6 cell lines were obtained from the American Type Culture Collection and cultured in Dulbecco’s modified Eagle’s medium (DMEM) and minimal essential medium (MEM), respectively, supplemented with 10% heat-inactivated fetal bovine serum (HI-FBS) (Thermo Fisher Scientific), 1% HEPES (Corning), 1% nonessential amino acids (Sigma-Aldrich), 1% sodium pyruvate (Sigma-Aldrich), and 2% penicillin-streptomycin mix (Thermo Fisher Scientific). Jurkat human T-lymphocyte lines were obtained from the American Type Culture Collection and cultured in RPMI 1640 (Thermo Fisher Scientific) supplemented with 10% HI-FBS (Thermo Fisher Scientific) and 1% HEPES (Corning).

### Isolation and culture of primary T lymphocytes.

Unidentified buffy coats, obtained from the blood of healthy adult donors according to a clinical protocol approved by the University of Texas Medical Branch at Galveston (UTMB) Institutional Review Board, were provided by the UTMB Blood Bank. Peripheral blood mononuclear cells (PBMCs) were isolated with a Histopaque (Sigma-Aldrich) gradient as recommended by the manufacturer. Fresh PBMCs were used for isolation of CD4^+^ and CD8^+^ T lymphocytes by negative selection using magnetic microbead separation kits (Miltenyi Biotec). “No-target” PBMCs were made devoid of monocytes and dendritic cells using positive selection for CD14 and CD11c to remove known EBOV target cells. Purity typically ranged from 93% to 95% as determined by flow cytometry. T-lymphocyte activation was induced with Dynabeads human transactivator CD3/CD28 beads (Thermo Fisher Scientific) according to the manufacturer’s recommendations. In some experiments, staphylococcal enterotoxin B (SEB; Sigma-Aldrich) was used as a control at a concentration of 1 μg/ml.

### Flow cytometry analysis of GP binding.

Isolated CD4^+^ T lymphocytes or Jurkat, 293T, or Vero-E6 cells were plated at the concentration of 1 × 10^6^ cells per well in U-bottom 96-well plates (Thermo Fisher Scientific) and placed on ice (to prevent internalization of viruses). EBOV was added at the indicated MOIs. Cells were incubated for 2 h at 4°C and washed with PBS containing 2% HI-FBS. Thereafter, cells were immunostained with the following antibodies: anti-CD3 labeled with brilliant UV 395 (BUV395) clone UCHT1 (BD Biosciences), anti-CD4 labeled with PerCP-Cy5.5 clone 4 Oktober (BioLegend) or anti-CD8-PerCP-Cy5.5 clone RPA-T8 (BioLegend), and rabbit antibodies raised against EBOV virus-like particles (VLP) (Integrated BioTherapeutics). For subset binding and depletion assays, the following antibodies were used in addition to the CD3 and CD4 antibodies previously described: CD38-Alexa.488 clone HIT2 (BioLegend), HLA-DR.APC clone L243 (BioLegend), CD45RO-BVLT.786 clone UCH-L1 (BioLegend), CCR7-PE clone G043H7 (BioLegend), and CXCR3-Alexa.Fluoro.700 clone 1C6/CXCR3 (BioLegend). After staining, cells were washed three times with PBS containing 2% HI-FBS, fixed in 10% formalin (Thermo Fisher Scientific), stained with goat anti-rabbit antibodies labeled with Alexa Fluor 647 (Thermo Fisher Scientific), and washed again 3 times in PBS with 2% HI-FBS. Following surface receptor staining, LIVE/DEAD (Thermo Fisher Scientific) staining was performed according to the manufacturer’s recommendation, and cells were fixed with 10% formalin. Flow cytometry was performed using a fluorescence-activated cell sorting (FACS) Fortessa (BD Biosciences) instrument.

### Analysis of markers of activation and cytokine analysis.

Following CD4^+^ T-cell isolation, cells were stimulated with EBOV at the indicated MOI for 48 h and surface stained for CD25-Alexa.Fluoro.700 clone M-A251 (BioLegend) and CD69-PE/Dazzle clone fn50 (BioLegend). Cells were fixed and permeabilized using the True Nuclear transcription factor kit (BioLegend) and stained for intracellular Ki-67-BVLT.421 clone B56 (BD Biosciences), IL-2–APC clone MQ1-17H12 (BD Biosciences), TNF-α–FITC clone Mab11 (BD Biosciences), and IFN-γ–PE clone B27 (BD Biosciences). Samples were then analyzed using a FACS Fortessa instrument (BD Biosciences). Cytokines and chemokines were detected and quantitated from supernatants by Eve Technologies (Calgary, Alberta, CA). Heat maps were generated using GENE-E (Broad Institute; http://www.broadinstitute.org/cancer/software/GENE-E/index.html).

### Intracellular accumulation of EBOV.

Viral binding assays were performed as described under “Flow cytometry analysis of GP binding” above with the additional step being performed prior to harvesting cell lysates. Following incubation on ice, cells were either immediately trypsinized or shifted to 37°C for 1 h to promote internalization of virions prior to trypsin digestion; nontrypsinized samples were used as a control. Cells were pelleted, washed in PBS, lysed as described under “BSL-4 work” above, and analyzed by Western blotting with antibodies specific to EBOV NP (IBT Bioservices; catalog no. 301-012), CD3ε (Santa Cruz Biotech; clone M-20), and glyceraldehyde-3-phosphate dehydrogenase (GAPDH) (Cell Signaling; clone 14C10). To further confirm reduction in TCR levels, EBOV stimulation was followed by Western blot analysis with antibodies specific for CD3ζ (Santa Cruz Biotech; clone 6B10.2).

### Analysis of NFAT-1 signaling.

CD4^+^ T cells or Jurkat T lymphocytes were plated at a concentration of 1 × 10^6^ cells per well in 96-well plates and mock treated or stimulated for 5, 10, 15, or 30 min with one of the following: 25 ng/ml 12-*O*-tetradecanoylphorbol-13-acetate (TPA), 0.5 μM ionomycin, 10 μM cyclosporine (CsA), 1 μg/ml SEB, or EBOV at an MOI of 5. Cells were collected, lysed in Laemmli buffer (Thermo Fisher Scientific), and incubated at 95°C for 15 min. Proteins were separated by SDS-PAGE using 4 to 12% gradient gels (Thermo Fisher Scientific) and transferred to nitrocellulose membranes (Thermo Fisher Scientific) using the I-blot system (Thermo Fisher Scientific). Membranes were blocked with 5% milk and 0.1% Tween 20 in PBS for 1 h at 37°C and stained with antibodies specific for mono- (activated) and hyperphosphorylated (nonactivated) NFAT-1 (Novus Biologicals, Inc.; catalog no. NB300-504) and GAPDH (Cell Signaling; catalog no. 8884S) diluted according to manufacturer’s recommendations in PBS with 0.1% Tween 20. Luciferase-based detection of NFAT activity was performed 24 h following electroporation of Jurkat cells with pGL3.NFAT-luciferase (78) (Addgene; catalog no. 17870) using the Neon transfection system per the manufacturer’s recommendation for the Jurkat cell line (Invitrogen). EBOV was added at an MOI of 3 while PMA-ionomycin was used as a positive control at 1 µg/ml each. Complete medium was added to control wells. Nontransfected Jurkat cells were used for background subtraction. Samples were harvested and lysed in 200 µl of passive lysis buffer 6 h following stimulation. Luciferase activity was determined using the luciferase assay system (Promega; catalog no. E1500). Data were normalized to control wells.

### Confocal microscopy.

Isolated primary CD4^+^ T cells were grown in suspension at 1 × 10^6^ cells/well in 12-well plates and loaded on positively charged coverslips (Thermo Fisher Scientific) for 2 h at 37°C. In some experiments, chloroquine (Sigma-Aldrich) was added to cell cultures at a concentration of 10 µM prior to the addition of EBOV. Cells were stimulated with EBOV at the indicated MOIs at 37°C, placed on ice, washed 3 times with PBS, and fixed with 3.2% paraformaldehyde for 15 min. Cells were permeabilized with 0.5% Triton X-100 (Alfa Aesar) solution in PBS for 15 min. Then, cells were washed with PBS, incubated with 0.5 M glycine in PBS for 30 min at room temperature, and washed 3 times with PBS. Antigen blocking was performed using 5% donkey serum diluted with PBS with 1% bovine serum albumin (BSA) and 0.1% Triton X-100 (stain buffer) for 1 h. Mouse monoclonal CD3ε, Rab7, and rabbit immune serum against EBOV VLP (Integrated BioTherapeutics) were diluted at 1:100 in stain buffer. After a 1-h incubation at room temperature, slides were washed 3 times in stain buffer, incubated with the mixture of two secondary antibodies (donkey anti-mouse conjugated with Alexa Fluor 488 [Thermo Fisher Scientific] and donkey anti-rabbit conjugated with Alexa Fluor 647 [Thermo Fisher Scientific]) diluted at 1:200 in stain buffer for 1 h, and washed as described above. Next, cells were incubated with 6-diamine-2-phenylindole-dihydrochloride (DAPI) (Thermo Fisher Scientific) at 1 µg/ml for 2 min and washed 3 times in PBS. Slides were then fixed in 4% paraformaldehyde and removed from BSL-4 as described above. Coverslips were mounted onto microscope slides using PermaFluor mounting medium (Thermo Fisher Scientific) and analyzed by laser scanning confocal microscopy using an Olympus FV1000 confocal microscope. Laser beams with 405-nm wavelengths were used for DAPI excitation, 488 nm was used for Alexa Fluor 488, and 635 nm was used for Alexa Fluor 647. Emission filters were 425/25 nm for DAPI, 515/30 nm for Alexa Fluor 488, and 610/50 nm for Alexa Fluor 647 detection. All images were acquired using a 60× oil objective.

### Phosphatidylserine-dependent binding assays.

Phosphatidylserine and phosphatidylcholine were obtained from Avanti Polar Lipids (Alabaster) and used to generate liposomes as previously described (79). CD4^+^ T cells were preincubated with liposomes at the indicated concentrations for 1 h prior to the addition of EBOV. Annexin V (EBioscience) was added to EBOV for 1 h prior to the addition to isolated CD4^+^ T cells. Flow cytometry analysis of EBOV GP was performed as described above.

Gene knockdown experiments were performed using pooled control or Tim-1-specific siRNA (Santa Cruz Biotech). Jurkat cells were electroporated using the Neon transfection system as recommended by the manufacturer (Invitrogen). Tim-1 levels were assessed by flow cytometry using anti-Tim-1–PE (R&D Systems; clone 219211). Assays were performed 48 h following electroporation using previously described culture conditions. The Lck inhibitor, Ly294, and PI3K inhibitor, PP2, were used at final concentrations of 10 µM (Sigma-Aldrich). Lysates for Western blot analysis were collected 2 h following EBOV or CD3/28 stimulation. Monoclonal rabbit antibodies for detecting Akt (clone C67E7), phospho-Akt (D9E), PI3K (19H8), and polyclonal phospho-PI3K were purchased from Cell Signaling Technologies.

### Transcriptome deep sequencing.

Isolated CD4^+^ T cells from four donors were cultured in RPMI 1640 medium in the presence or absence of EBOV at an MOI of 3 PFU/cell. At 24 and 96 h poststimulation, cells were washed with PBS 3 times, lysed in 1 ml of TRIzol (Thermo Fisher Scientific), and stored at −80°C. Samples were processed for RNA isolation using the Direct-zol RNA miniprep kit (Zymo Research) according to the manufacturer’s recommendations. RNA quality was assessed on an Agilent 2100 Bioanalyzer using the nanochip format, and only intact RNA was used for constructing the mRNA libraries. Libraries were constructed using the Kapa Stranded mRNA-Seq kit (Kapa Biosystems) according to the manufacturer’s instructions. Libraries were quality controlled and quantitated using the BioAnalyzer 2100 system and Qubit (Invitrogen). The libraries were clonally amplified and sequenced on an Illumina NextSeq 500 sequencer to achieve a target density of approximately 200,000 to 220,000 clusters/mm^2^ on the flow cell with dual-index paired-end sequencing at a 75-bp length using NextSeq 500 NCS v1.3 software. Raw reads (75 bp) had their adapter sequences removed.

### Read processing.

General quality control of the raw reads was performed using FastQC (http://www.bioinformatics.babraham.ac.uk/projects/fastqc/). rRNA reads were removed via mapping by Bowtie (v2.1.0) using an index of human, mouse, and rat rRNA sequences. On average, ~10% of the reads from each sample were identified as mapping to rRNA and were removed from downstream analysis. Reads were then mapped against a human reference genome (hg19, build GRCh37, from the UCSC genome browser [http://genome.ucsc.edu/]) using STAR (version 2.4.0h1). Quantitative gene counts were produced from this alignment using HTSeq (http://htseq.readthedocs.io/en/release_0.9.1/) utilizing the human annotation associated with the genome.

### Differential expression analysis.

Gene counts for 25,237 genes for each sample were loaded into the R statistical environment (http://www.r-project.org). Genes with no counts were removed, and counts across samples were normalized with edgeR (version 3.10.2) using the weighted trimmed mean of M values. Remaining genes without at least three samples with counts were removed, leaving 14,932 genes with an average of ~10^6^ reads per sample. Differentially expressed genes were identified using edgeR between treatments and time points and defined by using an absolute fold change cutoff of 1.5 and a *P* value of ≤0.05 after adjustment using the Benjamini-Hochberg multiple testing correction. Additional clustering, creation of heat maps, and other statistical analyses were performed using R. In the comparison between EBOV-treated samples and mock samples at day 1, 2,591 (1,581 upregulated, 1,010 downregulated) differentially expressed (DE) genes were identified. Between EBOV-treated and mock samples at day 4, 1,534 (789 upregulated, 745 downregulated) DE genes were identified.

### Functional enrichment analysis.

Functional analysis of the differential gene expression data was performed with Qiagen’s Ingenuity Pathway Analysis (IPA; Qiagen, Redwood City, CA). Functional annotation of genes for specific biological functions was assigned through querying AmiGO (version 2.20). Canonical pathway names were collected by searching for human genes with search queries as described in the text.

### Network analysis.

Functional analysis from IPA provided foundations for generating networks based on differentially expressed genes. DE genes with the most interactions with other DE genes were connected together to form networks with molecules from both the experimental expression data and IPA Knowledge databases. Each network was scored with a *P* value indicating its likelihood that these genes in the network could be found by random chance. IPA network scores of 2 or higher have at least a 99% confidence interval of not being generated by chance alone. The interactions within the network are based on IPA’s Knowledge Base and consist of direct and indirect relationships assigned based on primary literature sources. A top-scoring network (score of 41) with functions related to inflammation was used as the basis for the network depicting the cytokine storm response in Fig. 4F. A network (score of 33) with functions for cell cycle regulation was used in conjunction with its relationship with phosphatidylserine to form the phosphatidylserine regulation network in Fig. 5A. A network for cell signaling and interaction (score of 29) was used as a basis for a superantigen response (Fig. S3). Additionally, significant canonical pathways identified from IPA were assigned a *Z* score, which determines if gene expression changes from the expression data are consistent with the IPA Knowledge Base. Functions with a *Z* score of >2, indicating a strong predicted activation of that function, were chosen to form networks. Genes and interactions were additionally added or removed from these networks to highlight genes with the largest changes in expression and for clarity. Genes contributing to the construction of the cytokine storm networks were created from stricter absolute log 2-fold change cutoffs of >2× to focus on the interactions between the most highly activated genes.

### Statistical analyses.

Each independent experiment (donor) was performed in triplicate to rule out experimental bias or random error. Data were analyzed using statistical methods described in the figure legends using GraphPad Prism 6. *P* values of <0.05 were considered statistically significant. Mean and standard error (SE) of the mean were calculated for all graphs.
