# Supplementary material for: Ebola Virus Binding to Tim-1 on T Lymphocytes Induces a Cytokine Storm
Source: mBio. 2017 Sep 26;8(5):e00845-17. doi: 10.1128/mBio.00845-17 (PMC5615193; doi:10.1128/mBio.00845-17)
Supplement: TABLE S1 [file mbo005173494st1.docx]

**Table S1.** Analysis of cytokines and chemokines in sera of wild type and Tim-1^-/-^ mice infected with EBOV

| **Cytokine/**  **Chemokine** | **wt mice**  **mock** | **wt mice**  **EBOV** | **Tim-1^-/-^ mice**  **EBOV** | **P-value** |  |
| --- | --- | --- | --- | --- | --- |
| CCL2 | 14.6 ± 3.5 | 7738.6 ± 1969.3 | 4952.9 ± 2198.1 | 0.378 | |
| CCL3 | 30.9 ± 5.9 | 437.2 ± 43.7 | 222.8 ± 34.1 | 0.008* | |
| CCL4 | 26.4 ± 3.4 | 894.2 ± 207.0 | 789.6 ± 175.4 | 0.720 | |
| CCL5 | 15.2 ± 1.3 | 224.3 ± 42.0 | 87.2 ± 20.8 | 0.031* | |
| CXCL1 | 54.7 ± 11.9 | 341.1 ± 35.0 | 407.6 ± 118.6 | 0.569 | |
| CXCL2 | 240.2 ± 22.7 | 408.2 ± 111.6 | 301.4 ± 21.4 | 0.431 | |
| CXCL5 | 7187.9 ± 1157.8 | 1382.6 ± 306.8 | 174.1 ± 66.8 | 0.011* | |
| CXCL9 | 62.9 ± 7.4 | 1455.2 ± 214.7 | 1110.8 ± 189.0 | 0.281 | |
| CXCL10 | 56.7 ± 8.5 | 3831.9 ± 746.5 | 3471.7 ± 925.7 | 0.768 | |
| Eotaxin | 553.2 ± 42.6 | 750.8 ± 37.9 | 734.5 ± 21.2 | 0.738 | |
| G-CSF | 149.5 ± 12.1 | 1467.2 ± 252.3 | 3036.2 ± 410.3 | 0.011* | |
| GM-CSF | 17.9 ± 1.5 | 22.4 ± 1.8 | 16.8 ± 0.6 | 0.033* | |
| IFNγ | <1.0 ± 0.0 | 472.7 ± 65.4 | 203.8 ± 20.2 | 0.009* | |
| IL1α | 260.3 ± 50.1 | 181.9 ± 11.5 | 201.3 ± 10.8 | 0.354 | |
| IL1β | 13.1 ± 0.8 | 22.7 ± 3.9 | 18.4 ± 3.5 | 0.456 | |
| IL2 | 5.0 ± 0.7 | 21.6 ± 4.0 | 8.2 ± 1.6 | 0.026* | |
| IL3 | 0 ± 0.0 | 0.9 ± 0.3 | 1.1 ± 0.2 | 0.603 | |
| IL4 | 0.2 ± 0.1 | 0.4 ± 0.1 | 0.2 ± 0.1 | 0.046* | |
| IL5 | 6.1 ± 0.9 | 7.2 ± 2.0 | 5.1 ± 0.6 | 0.396 | |
| IL6 | 0.8 ± 0.3 | 190.7 ± 33.5 | 443.9 ± 94.2 | 0.027* | |
| IL7 | 1.4 ± 0.3 | 38.6 ± 3.1 | 21.1 ± 6.9 | 0.041* | |
| IL9 | 12.4 ± 1.8 | 27.2 ± 1.9 | 23.9 ± 3.0 | 0.371 | |
| IL10 | 2.3 ± 0.2 | 195.5 ± 35.1 | 68.6 ± 18.1 | 0.021* | |
| IL12p40 | 11.6 ± 5.7 | 2.6 ± 0.6 | 27.6 ± 5.8 | 0.002* | |
| IL12p70 | 5.2 ± 0.8 | 9.5 ± 1.7 | 21.4 ± 4.5 | 0.030* | |
| IL13 | 17.1 ± 4.2 | 52.7 ± 2.6 | 53.2 ± 9.3 | 0.962 | |
| IL15 | 6.8 ± 2.0 | 88.2 ± 11.8 | 36.0 ± 12.7 | 0.020* | |
| IL17 | 0.1 ± 0.1 | 1.5 ± 0.4 | 2.1 ± 0.5 | 0.402 | |
| LIF | 0.0 ± 0.0 | 19.7 ± 1.6 | 35.5 ± 5.7 | 0.021* | |
| M-CSF | 7.8 ± 0.5 | 14.6 ± 1.2 | 12.7 ± 0.7 | 0.258 | |
| TNFα | 2.5 ± 0.8 | 64.9 ± 7.6 | 41.3 ± 3.7 | 0.038* | |
| VEGF | 0.7 ± 0.2 | 0.5 ± 0.1 | 0.5 ± 0.1 | 0.886 | |

Serum cytokine and chemokine levels were determined using multiplex analysis at day 6 post EBOV infection. Results shown are average of 4 mice for the Tim-1^-/-^ group and 5 mice for the mock and wild type control groups ± SE. P-values are indicated for Tim-1^-/-^ versus control, EBOV-infected wild type CD57bl/6 mice. * P<0.05.
