# Supplementary figures and images for: Ebola Virus Binding to Tim-1 on T Lymphocytes Induces a Cytokine Storm
Source: mBio. 2017 Sep 26;8(5):e00845-17. doi: 10.1128/mBio.00845-17 (PMC5615193; doi:10.1128/mBio.00845-17)

**Fig. S1**

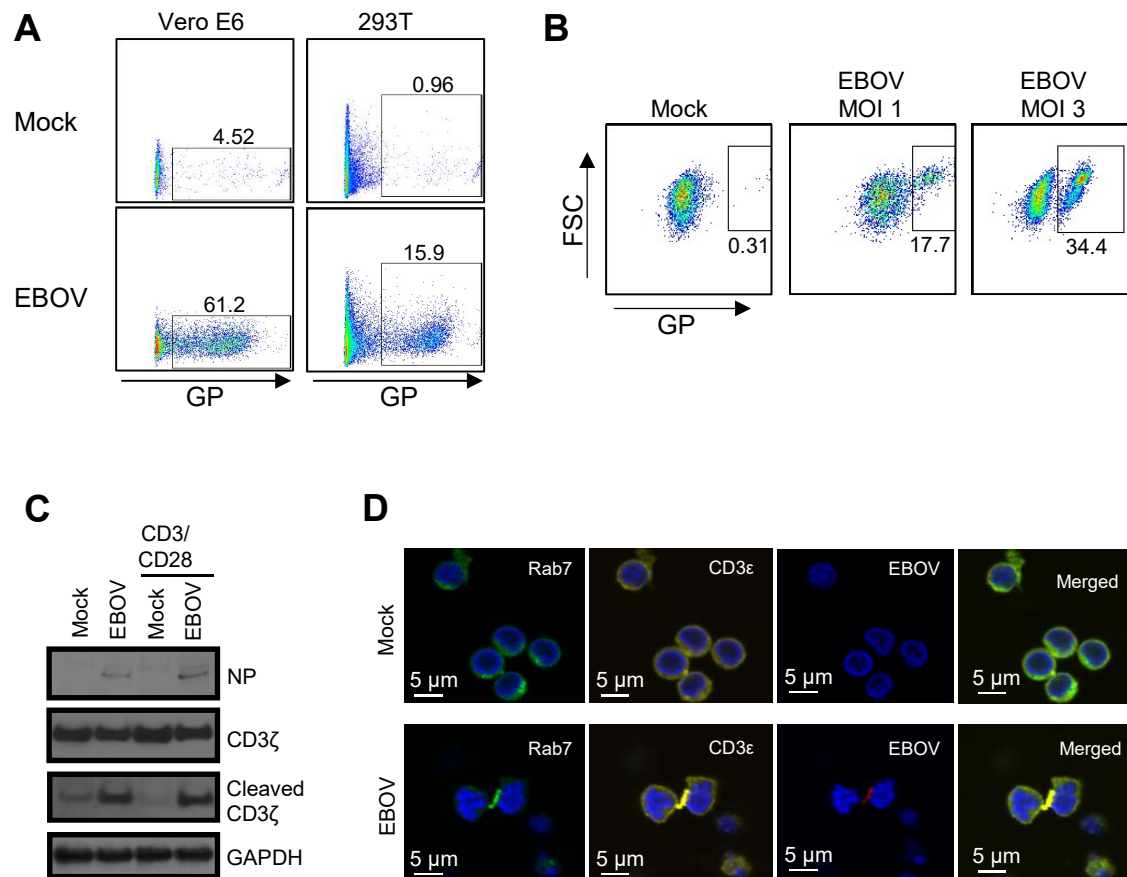

Supplement: FIG S1 [file mbo005173494sf1.pdf]

**Fig. S2**

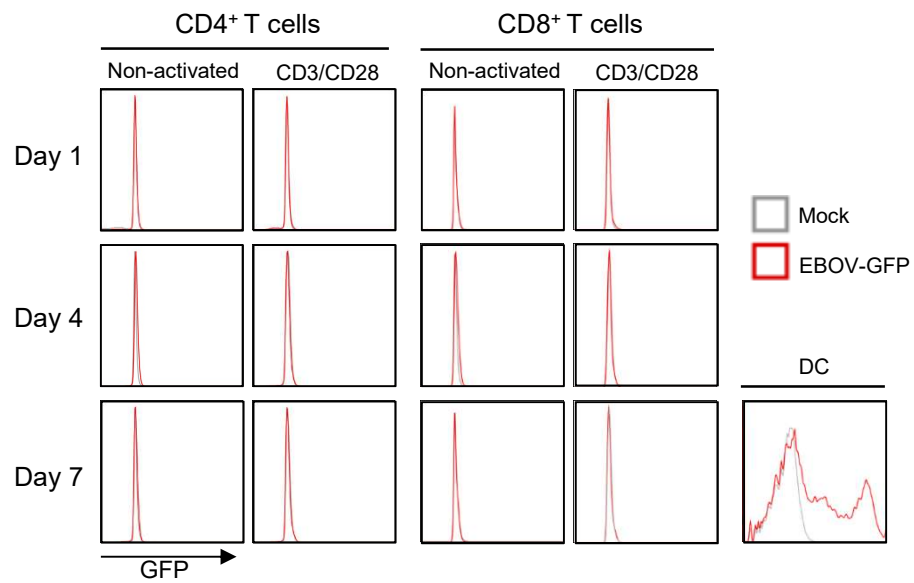

Supplement: FIG S2 [file mbo005173494sf2.pdf]

Fig. S3

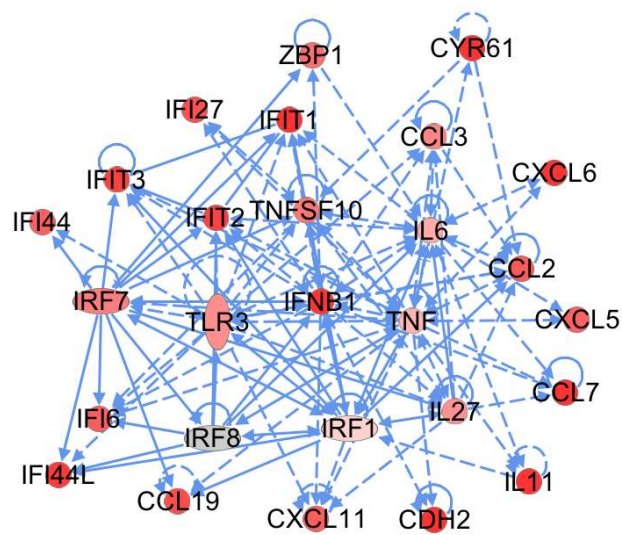

Supplement: FIG S3 [file mbo005173494sf3.pdf]

**Fig. S4**

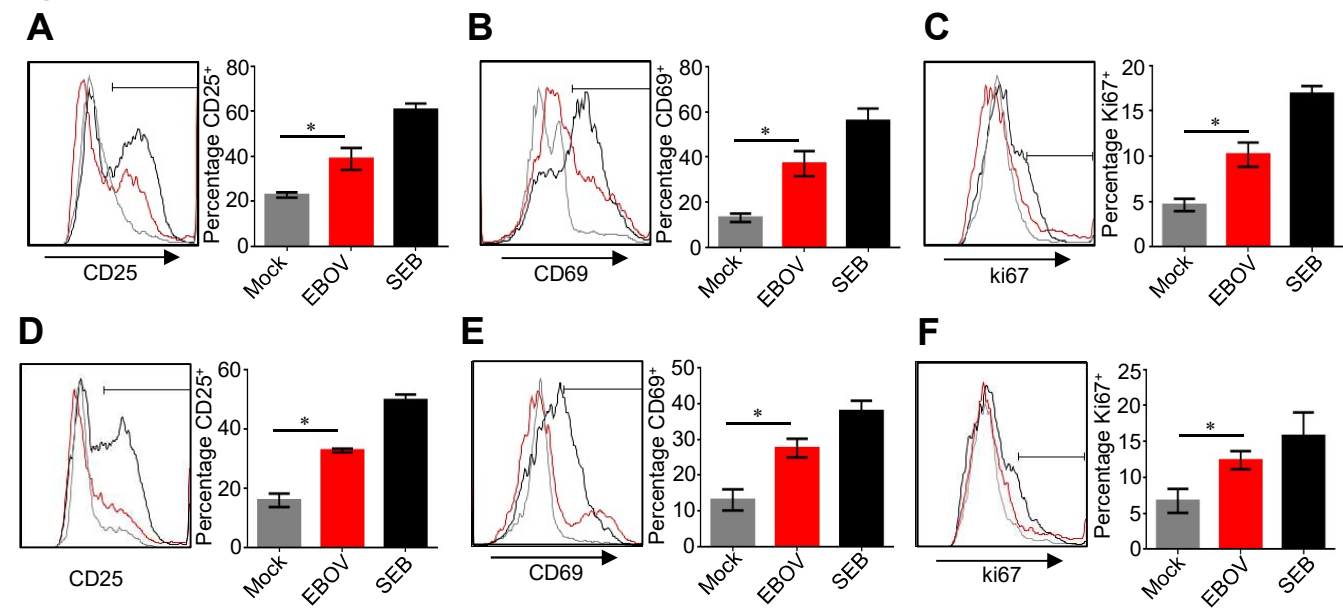

Supplement: FIG S4 [file mbo005173494sf4.pdf]

**Fig. S5**

**A**

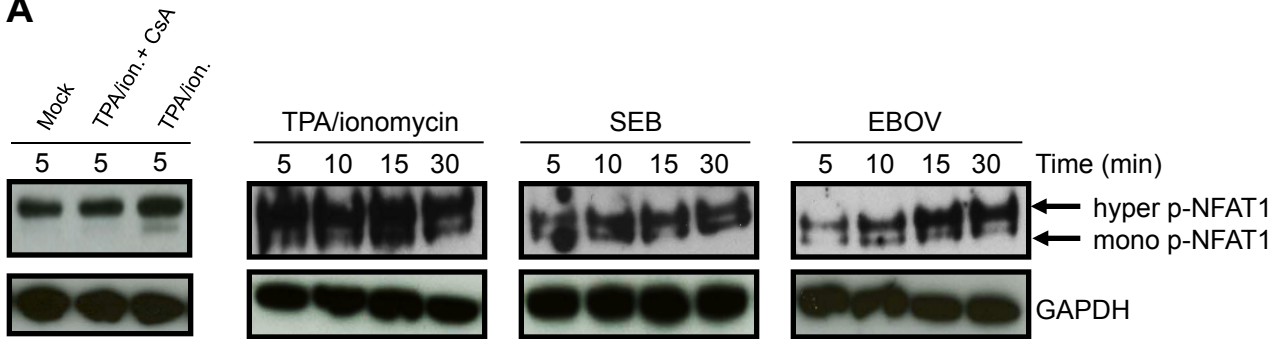

**B**

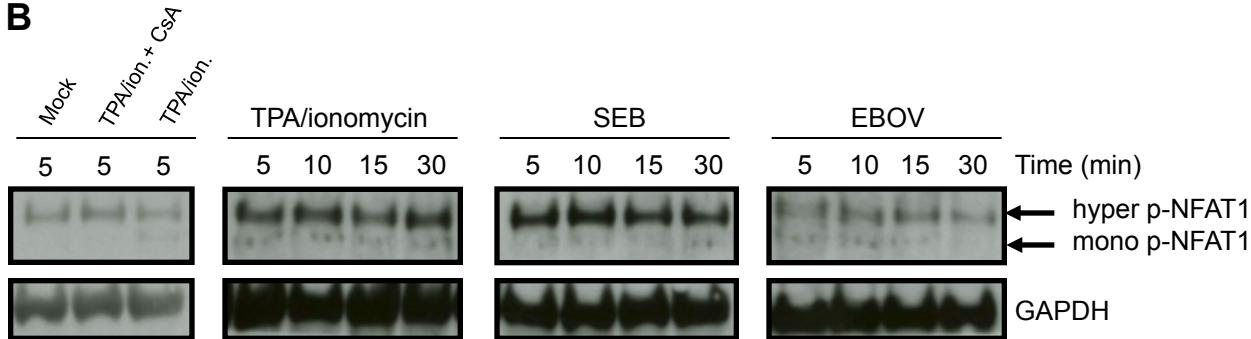

**C**

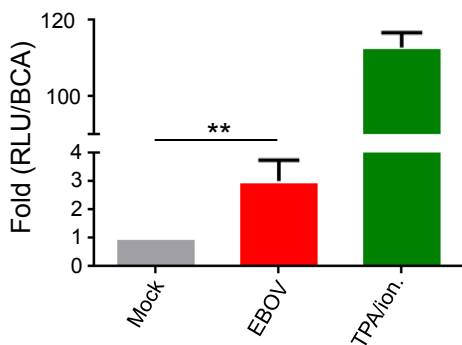

Supplement: FIG S5 [file mbo005173494sf5.pdf]

Fig. S6

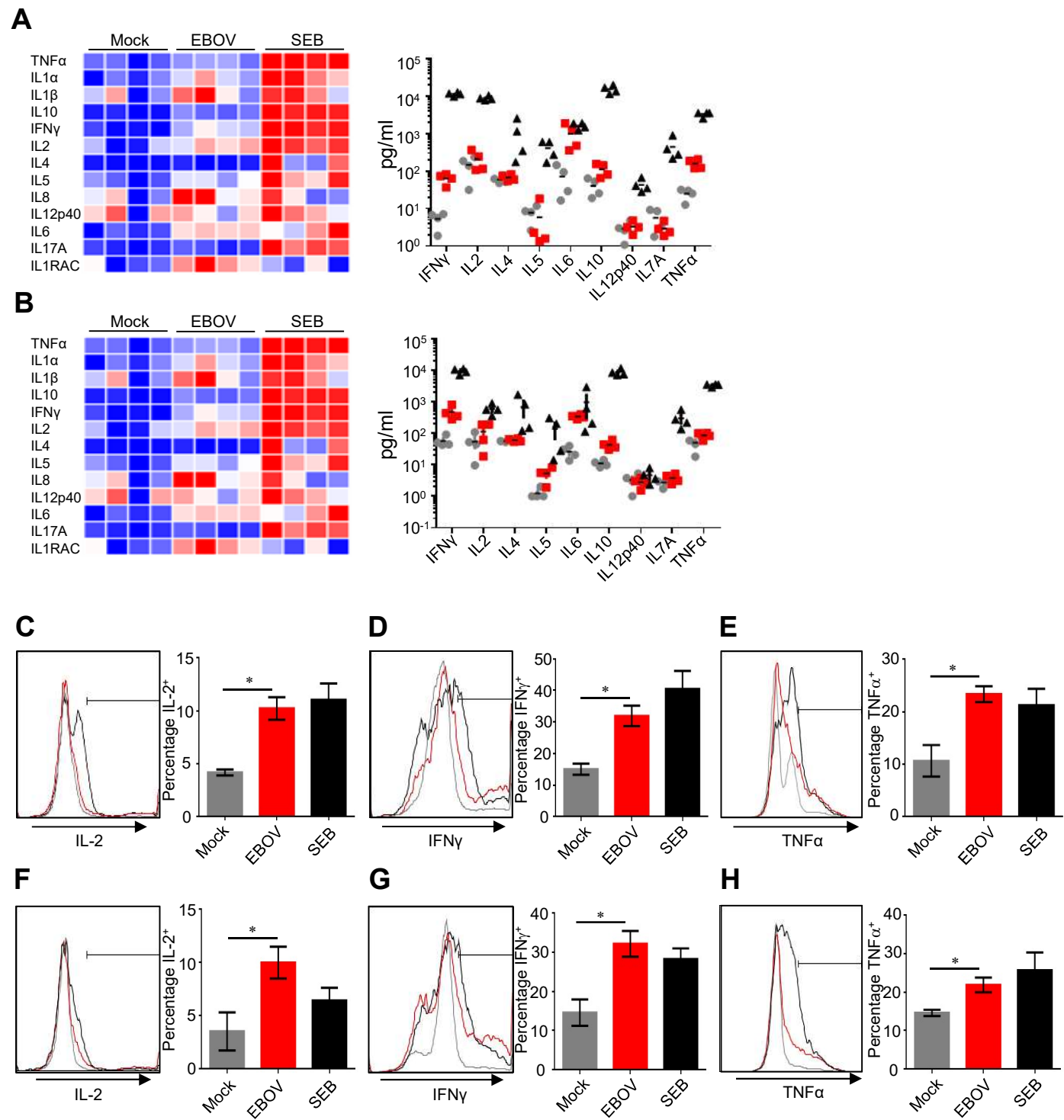

Supplement: FIG S6 [file mbo005173494sf6.pdf]

**Fig. S7**

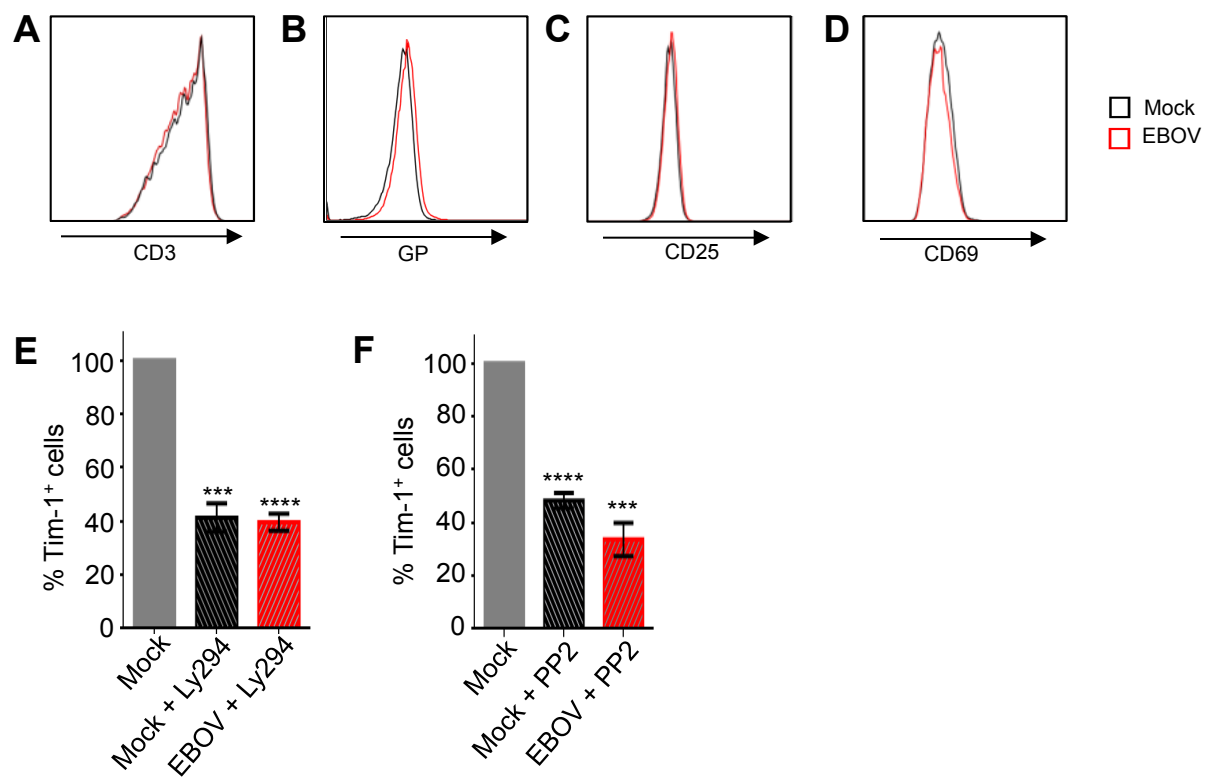

Supplement: FIG S7 [file mbo005173494sf7.pdf]

**Fig. S8**

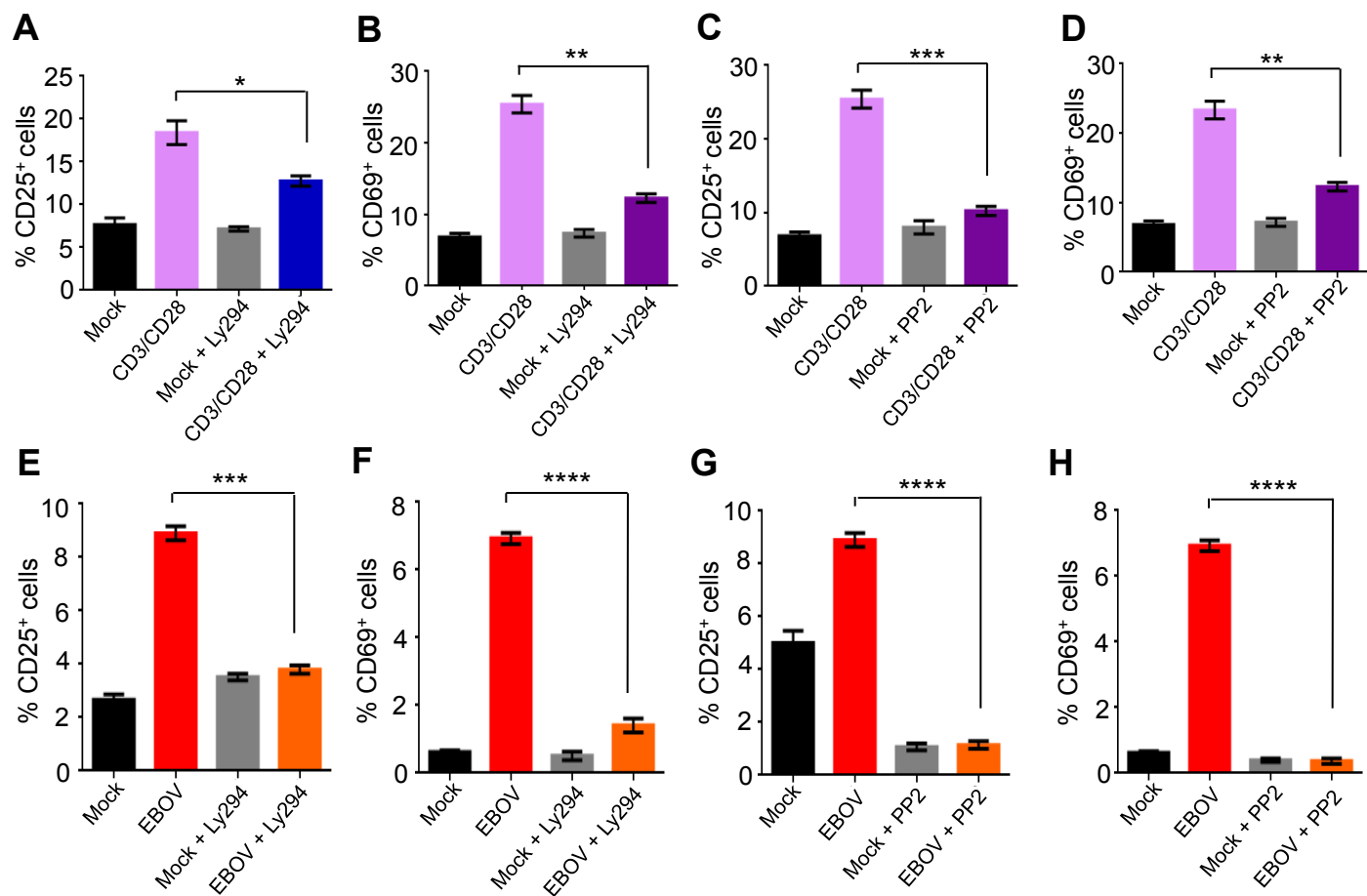

Supplement: FIG S8 [file mbo005173494sf8.pdf]
