# Supplementary material for: Ebola Virus Binding to Tim-1 on T Lymphocytes Induces a Cytokine Storm
Source: mBio. 2017 Sep 26;8(5):e00845-17. doi: 10.1128/mBio.00845-17 (PMC5615193; doi:10.1128/mBio.00845-17)
Supplement: TABLE S2 [file mbo005173494st2.docx]

**Table S2.** Cytokine response of immune cells to EBOV

| **Cytokine** | **PBMCs,**  **fold change**  **over mock** | **P-value** | **PBMC**  **(no target), fold change over mock** | **P-value** | **CD4^+^ T-cells alone, fold change over mock** | **P-value** |
| --- | --- | --- | --- | --- | --- | --- |
| IFNγ | 12.0* | 0.001 | 8.1* | 0.012 | 33.8* | 0.004 |
| IL2 | 1.4 | 0.456 | 2.1 | 0.257 | 5.3 | 0.003 |
| IL4 | 1.1 | 0.391 | 1.1 | 0.304 | 1.2 | 0.209 |
| IL5 | -1.3 | 0.701 | 4.2* | 0.022 | 6.2* | 0.001 |
| IL6 | 13.9* | 0.042 | 12.8* | <0.001 | 91.2* | <0.001 |
| IL10 | 2.8* | 0.028 | 3.9* | 0.004 | 15.6* | 0.001 |
| IL12p40 | 1.1 | 0.676 | -1.2 | 0.622 | 2.5* | 0.014 |
| IL17A | -1.9 | 0.210 | 1.4 | 0.212 | 2.8* | 0.088 |
| TNFα | 6.3* | 0.001 | 1.7 | 0.065 | 6.8* | 0.037 |

Comparative analysis of cytokine levels on day 4 of culture of PBMC, PBMC with monocytes and DC depleted (no target), and purified CD4^+^ T cells in the presence of EBOV at an MOI of 1 PFU/cell. Mean fold change, compared to mock, based on PBMC from 4 donors. * Significant changes, *P*<0.05 (Student T-test).
